# Study protocol for a randomised, phase II, double-blind, experimental medicine study of obinutuzumab versus rituximab in ANCA-associated vasculitis: ObiVas

**DOI:** 10.1136/bmjopen-2023-083277

**Published:** 2024-07-17

**Authors:** Dominic Paul McGovern, Mark E McClure, Matthew Coates, Simon Bond, Marcos Martinez Del Pero, Kim Mynard, Jacinta Lee, Rona M Smith, David R Jayne, Menna Ruth Clatworthy, Rachel B Jones, on behalf of the ObiVas Investigators

**Affiliations:** 1 Department of Medicine, University of Cambridge, Cambridge, UK; 2 Vasculitis and Lupus Clinic, Addenbrooke’s Hospital, Cambridge University Hospitals NHS Foundation Trust, Cambridge, UK; 3 Cambridge Clinical Trials Unit, Cambridge University Hospitals NHS Foundation Trust, Cambridge, UK; 4 Cambridge University Hospitals NHS Foundation Trust, Cambridge, UK; 5 Faculty of Cellular Genetics, Wellcome Sanger Institute, Hinxton, UK

**Keywords:** immunology, nephrology, otolaryngology, clinical trials

## Abstract

**Introduction:**

Relapses in ANCA-associated vasculitis (AAV) increase the incidence of end-organ damage and their prevention requires prolonged immunosuppressive therapy. Rituximab, a type I anti-CD20 B cell depleting monoclonal antibody, is the current standard of care for induction of disease remission. Rituximab is not always effective and is associated with a high subsequent relapse risk. Obinutuzumab is a type II anti-CD20 humanised monoclonal antibody with the potential to obtain greater tissue B cell depletion than rituximab and reduce relapse risk in AAV.

**Methods and analysis:**

ObiVas is a randomised, phase II, double-blind controlled trial that will compare the mechanistic effects of rituximab and obinutuzumab in the induction treatment of patients with AAV positive for proteinase 3 ANCA (PR3-ANCA). 26 patients, either newly diagnosed or relapsing, will be recruited from a single centre and randomised in a 1:1 ratio to receive 1000 mg rituximab or obinutuzumab as induction therapy on days 1 and 15, alongside a tapering glucocorticoid regimen. The primary end point is CD19^+^ B cell depletion in nasal-associated lymphoid tissue (NALT), assessed as change from baseline to week 26. Secondary outcomes will compare the safety and clinical efficacy of rituximab and obinutuzumab and their impact on immune biomarkers, including tissue and peripheral blood lymphocyte subsets and PR3-ANCA binding levels. Patients are followed through to week 78. The trial opened for recruitment in January 2023 and is forecasted to complete recruitment by the end of 2024.

**Ethics and dissemination:**

For all patients, informed written consent will be obtained in keeping with Good Clinical Practice. Trial results will be disseminated to the relevant scientific, clinical and patient communities on trial closure. NALT data analysis will start before trial completion. Other analyses will be reported after trial completion. This trial was given ethical approval by Edgbaston (West Midlands) Research Ethics Committee (approval reference 22/WM/0174).

**Trial registration number:**

ISRCTN13069630.

**STRENGTHS AND LIMITATIONS OF THIS STUDY**

- Experimental medicine study with a biomarker primary objective, in addition to multiple secondary and exploratory objectives of translational interest.
- Trial uses unique methodology of sampling nasal-associated lymphoid tissue, offering a window into how B cell depleting therapies impact tissue immunity.
- In-depth profiling of participants’ immunobiology, including single-cell RNA sequencing, aims to provide novel insights into ANCA-associated vasculitis pathogenesis and treatment response.
- Single tertiary centre study with previous experience in these end points and conducting vasculitis trial.
- The trial is not powered to test for clinical efficacy or safety, but a positive primary end point would provide human in vivo evidence for larger-scale trials.

## Introduction

### Background and rationale

 ANCA-associated vasculitis (AAV) is a group of primary vasculitides, characterised by the presence of autoantibodies against the neutrophil antigens proteinase 3 (PR3-ANCA) or myeloperoxidase (MPO-ANCA) and leucocyte infiltration of small blood vessel walls, resulting in vascular damage and organ dysfunction. Patients with vasculitis with PR3-ANCA are commonly diagnosed with granulomatosis with polyangiitis (GPA), characterised by small vessel vasculitis and inflammation, primarily affecting the upper and lower respiratory tract and the kidneys. Alongside glucocorticoids, cyclophosphamide or the B cell depleting anti-CD20 monoclonal antibody (mAb), rituximab, are both standard remission induction therapies and rituximab is favoured over cyclophosphamide in relapsing GPA. The complement C5a receptor antagonist avacopan was approved in the UK in 2022 and can be used in clinical practice as a glucocorticoid-sparing therapy in severe active AAV.

The rationale for targeting B cells in ANCA vasculitis is supported by studies demonstrating activated B cells in inflammatory lesions, the association of B cell activation status with disease activity and the efficacy of rituximab.^1^,^4^ However, the RAVE (rituximab in ANCA-associated vasculitis) trial demonstrated that a single course of rituximab as induction remission, without maintenance therapy, is associated with a 60% relapse rate at 18 months.^5^ Relapses usually occur after the return of peripheral blood B cells.^3^ Patients positive for PR3-ANCA are at higher risk of relapsing than those who are seronegative.^6^ This risk of relapse means that maintenance of immunosuppressive therapy—commonly 4–6 monthly interval doses of rituximab—is usually required, which adds to the burden of drug toxicity and is associated with long-term complications such as hypogammaglobulinaemia and infection.^7^

There are several explanations for why AAV commonly relapses following rituximab. Studies in rheumatoid arthritis and kidney transplantation (and also in AAV, unpublished data collected by our group), have demonstrated that while rituximab depletes peripheral B cells almost completely, tissue B cell subsets persist, suggesting the ability of rituximab to penetrate tissue is limited.^8 9^ There is strong circumstantial evidence that persistence of tissue B cells are associated with relapse.^10^ Furthermore, the internalisation of the rituximab-CD20 complex (and its removal from the cell surface by myeloid cells—a process known as trogocytosis) may also reduce the half-life of rituximab.^11^

Obinutuzumab is a newer IgG1, humanised, glycoengineered type II mAb against CD20. Relative to rituximab, it has been shown to exhibit increased direct and immune effector cell-mediated cytotoxicity. In primate studies, obinutuzumab has demonstrated superior tissue B cell depletion, including in lymphoid tissue.^12^ The affinity of the Fc region of obinutuzumab has higher binding affinity to the FcγRIII expressed on effector cells, enhancing antibody-dependent cellular cytotoxicity and phagocytosis.^11^ The obinutuzumab-CD20 complexes are also less likely to be internalised by the targeted B cells (or trogocytosed by myeloid cells) compared with the equivalent rituximab-CD20 complexes, increasing the ability of obinutuzumab to recruit effector cells.^13^ Thus far, clinical trials have demonstrated that obinutuzumab is more efficacious than rituximab in treating chronic lymphocytic leukaemia (phase III), and effective compared with placebo in lupus nephritis (phase II).^14 15^

We hypothesise that obinutuzumab will deplete tissue-resident B cells in ANCA-associated vasculitis (AAV) to a greater extent than rituximab. We hypothesise that superior tissue B cell depletion will delay peripheral blood B cell reconstitution, with potential to improve clinical outcomes such as sustained remission in AAV.

## Methods and analysis

### Trial objectives

#### Primary objective

To compare the differential effects of obinutuzumab versus rituximab to deplete nasal tissue B cells in AAV.

#### Secondary objective: efficacy (biomarkers)

- To compare the differential effects of obinutuzumab versus rituximab on nasal tissue B and T cell subsets.
- To compare the differential effects of obinutuzumab versus rituximab on blood B and T cell subsets.
- To compare the differential effects of obinutuzumab versus rituximab on B cell depletion and reconstitution in blood.
- To compare the differential effects of obinutuzumab versus rituximab on changes in PR3 ANCA.

#### Secondary objective: clinical efficacy/safety

- To compare the clinical efficacy of obinutuzumab versus rituximab.
- To compare the safety of obinutuzumab versus rituximab.

#### Exploratory objectives

- To compare the differential effects of obinutuzumab versus rituximab on T-B cell cross-talk in nasal tissue.
- To compare the differential effects of obinutuzumab versus rituximab on B cell receptor (BCR) repertoire in tissue and blood.
- To compare the differential effects of obinutuzumab versus rituximab on functionality of B and T cell in the blood.

### Trial design

ObiVas is a single-centre, experimental medicine, randomised, phase II, double-blind, controlled trial designed to evaluate the mechanistic effect of obinutuzumab versus rituximab in severe newly diagnosed or relapsing PR3-ANCA positive AAV. Participants will be randomised to one of two treatment groups in a 1:1 ratio and receive: obinutuzumab (2×1000 mg, 2 weeks apart) plus prednisolone; or rituximab (2×1000 mg, 2 weeks apart) plus prednisolone. The oral prednisolone tapering regimens will be identical in both groups. All participants will undergo nasal biopsies at baseline and week 26, to allow for the assessment of the primary end point—comparison of change from baseline of CD19^+^ B cells in the nasal-associated lymphoid tissue (NALT). Follow-up will last for 78 weeks following entry into the study and adherence to the trial protocol (in particular the protocolised steroid taper) will be reviewed at each visit. 26 participants will be randomised and dosed with obinutuzumab or rituximab. Primary end point analysis will be performed after all participants have completed the week 26 biopsy and assessment. The second NALT biopsy will be performed at week 26, when rituximab is no longer detectable in blood.

### Interventions

Those randomised to receive rituximab will receive 1000 mg on both day 1 and day 15. Likewise, those randomised to receive obinutuzumab will receive 1000 mg on both day 1 and day 15. This rituximab dosing is the recommended induction regimen in the UK.^16^ The obinutuzumab dosing is the same as the induction dosing used in clinical trials of systemic lupus erythematosus and lupus nephritis.^15 17 18^ Both investigational medicinal products (IMPs) are administered using the same infusion volume, rate, duration and premedication regimen. Blinding is therefore straightforward.

The upper respiratory tract is a disease relevant site, particularly in patients with PR3 AAV, in whom sino-nasal granulomatous inflammation is common. Our team, using in-house ear, nose and throat (ENT) expertise, has developed a local anaesthetic biopsy technique to sample NALT. The procedure is well tolerated with no serious complications reported to date, while producing good cell yields (median 250 000 cells per biopsy, of which the majority are immune cells). The dropout rate for participants involved in sequential procedures is low (<5%).

A NALT biopsy will be performed on all participants at baseline (on or prior to day 1, before IMP administration) and again at week 26. These will be performed by an experienced ENT surgeon, under local anaesthetic, which allow cross-compartment (blood, tissue, urine) analyses and change from baseline comparisons—including the primary end point assessment of CD19^+^ B cells in lymphoid tissue, quantified by flow cytometry.

### Sample size

In patients with AAV, rituximab achieves 90%–95% peripheral blood CD19^+^ B cell depletion assessed by flow cytometry. However, tissue CD19^+^ depletion with rituximab is lower, estimated at 55% based on our own previous NALT studies in six patients with AAV taken 3–6 months after rituximab treatment (unpublished). Obinutuzumab depleted peripheral B cells effectively in patients with lupus nephritis^15^ and preclinical data of obinutuzumab in cynomolgus monkeys found reduction in tissue B cells of 90%–95%.^3^ ObiVas is the first human study to assess tissue B cell depletion with obinutuzumab in autoimmune disease. Based on these factors, we have predicted a conservative estimate of 80% CD19^+^ B cell depletion following obinutuzumab.

For design calculations, we considered mean values of 55% CD19^+^ B cell depletion in the rituximab arm, and 75% and 80% CD19^+^ B cell depletion in the obinutuzumab arm, with an assumed common SD of 14.5%. The design calculations are shown in table 1.

**Table 1:** : Sample Size Calculation

Sample size	55% RTX vs 75% OBI CD19^+^ B cell depletion	55% RTX vs 80% OBI CD19^+^ B cell depletion
N=20	83% power	95% power
N=26	92% power	99% power

OBIobinutuzumabRTXrituximab

In addition, based on prior experience it is anticipated that 10% (2–3 participants) will have treatment failure in the first 6 months with a postrandomisation withdrawal rate of <5% (0–1 participants). Inadequate nasal sample acquisition rate is also expected to be low at <5% (0–1 participant). Therefore, a per-protocol (PP) end point analysis will consist of 20–26 participants.

### Study setting

All participants will be recruited from the vasculitis service at Addenbrooke’s Hospital, Cambridge University Hospitals NHS Foundation Trust (UK).

### Patient and public involvement

The Vasculitis and Lupus Clinic at Addenbrooke’s Hospital has conducted previous clinical trials in AAV. Patient feedback from the COMBIVAS (a randomised study of rituximab and belimumab sequential therapy in PR3 ANCA-associated vasculitis) trial,^19^ in which patients also had paired NALT biopsies, helped inform the ObiVas trial design. Furthermore, we previously collected patient questionnaires on feasibility and tolerability of the nasal biopsy technique. Results will be published in medical journals, fed back to participants directly as well as to Vasculitis UK—the national patient-led charity—for dissemination to the wider vasculitis patient community.

### Eligibility criteria

To be eligible, participants must be 18 years of age or older and capable of giving informed consent. They must have a diagnosis of AAV (GPA or microscopic polyangiitis) according to the definitions of the Chapel Hill Consensus Conference, a positive PR3-ANCA (by ELISA) at screening and active disease (either newly diagnosed or relapsing), defined by one major or three minor disease activity items on the Birmingham Vasculitis Activity Score for Wegener’s (BVAS/WG). All participants must have received at least two vaccines for COVID-19 or have detectable COVID-19 antibodies and women of childbearing potential must agree to use effective contraception, until at least 18 months after the second and final protocolised dose of rituximab/obinutuzumab.

Key exclusion criteria include a positive MPO-ANCA or anti-GBM (glomerular basement membrane) antibodies at screening, an estimated glomerular filtration rate <15 mL/min/1.73 m^2^ or the presence of pulmonary haemorrhage with hypoxia. Patients with significant hypogammaglobulinaemia (IgG <400 mg/dL), neutropaenia (<1.5×10^9^ cells/L) and B cell lymphopaenia (total blood CD19^+^ count <0.1×10^9^/L) at screening will be excluded. Those who have suffered from malignant neoplasms within 5 years will also be excluded (with the exceptions of non-melanoma skin and uterine cancers, providing they have been treated locally without metastatic disease). Any patients who have received: rituximab in the preceding 12 months; cyclophosphamide in the preceding 6 months or plasmapheresis/intravenous immunoglobulin in the preceding 90 days from day 1 will be excluded. Participants will be allowed no more than 3 g of emergency intravenous methylprednisolone between 30 days prior to screening and day 1. Participants will also be screened for infection (including bloodborne viruses and tuberculosis). A full listing of the inclusion and exclusion criteria are provided in online supplemental appendix 1.

### Outcomes

The primary outcome of ObiVas is the relative percentage change in NALT CD19^+^ B cells between baseline and week 26 in both treatment arms, measured by spectral flow cytometry. We hypothesise that obinutuzumab will be more efficacious in the depletion of tissue-resident B cells than rituximab and that, consequently, this will prolong peripheral B cell depletion; reduce differentiation into ANCA-producing plasma cells (impacting circulating ANCA titres) and reduce the antigen-presenting capabilities of B cells to autoreactive T cells, thus reducing activation of the lymphoid T cell compartment. Clinically, we hypothesise that there will be a longer time to relapse in those who have received obinutuzumab.

#### Primary outcome

- Relative percentage change in live single nasal CD19^+^ B cells in the nasal lymphoid compartment between baseline and week 26 (spectral flow cytometry)

#### Secondary outcomes

- Relative percentage change in nasal B and T cell subsets between baseline and week 26 (spectral flow cytometry—antigens of interest will include CD19, CD20, CD24, CD27, CD28, CD38, CD45, CD127, CCR5-7, CXCR3, CXCR5, IgM and IgD)
- Relative percentage change from baseline in blood B, T, NK cells and subsets of interest at weeks 12, 26, 39, 52, 65 and 78 (spectral flow cytometry—antigens of interest as above, plus CD56, CD57 and CD69)
- Incidence of participants with detectable peripheral B cells at weeks 12, 26, 39, 52, 65 and 78 (spectral flow cytometry—antigens of interest as above).
- Incidence of participants with PR3-ANCA negativity at weeks 12, 26, 39, 52, 65 and 78 (ELISA).
- Time to PR3-ANCA rise (ELISA).
- Incidence of participants in sustained remission (relapse-free) at weeks 12, 26, 39, 52, 65 and 78 (remission defined as BVAS-WG ≤1 with daily prednisone dose ≤7.5 mg).
- Time to remission (BVAS-WG).
- Time to first relapse in those who have achieved remission (BVAS-WG).
- Time to first major or second minor relapse in those who have achieved remission (BVAS-WG).
- Cumulative exposure to corticosteroids between groups (steroid dose calculations).
- Incidence of participants with serious adverse events (SAEs) at week 78 (SAE reporting).
- Incidence of SAEs (SAE reporting).
- Incidence and severity of adverse events of special interest (AESIs, AESI reporting).

#### Exploratory outcomes

**Figure 1:**
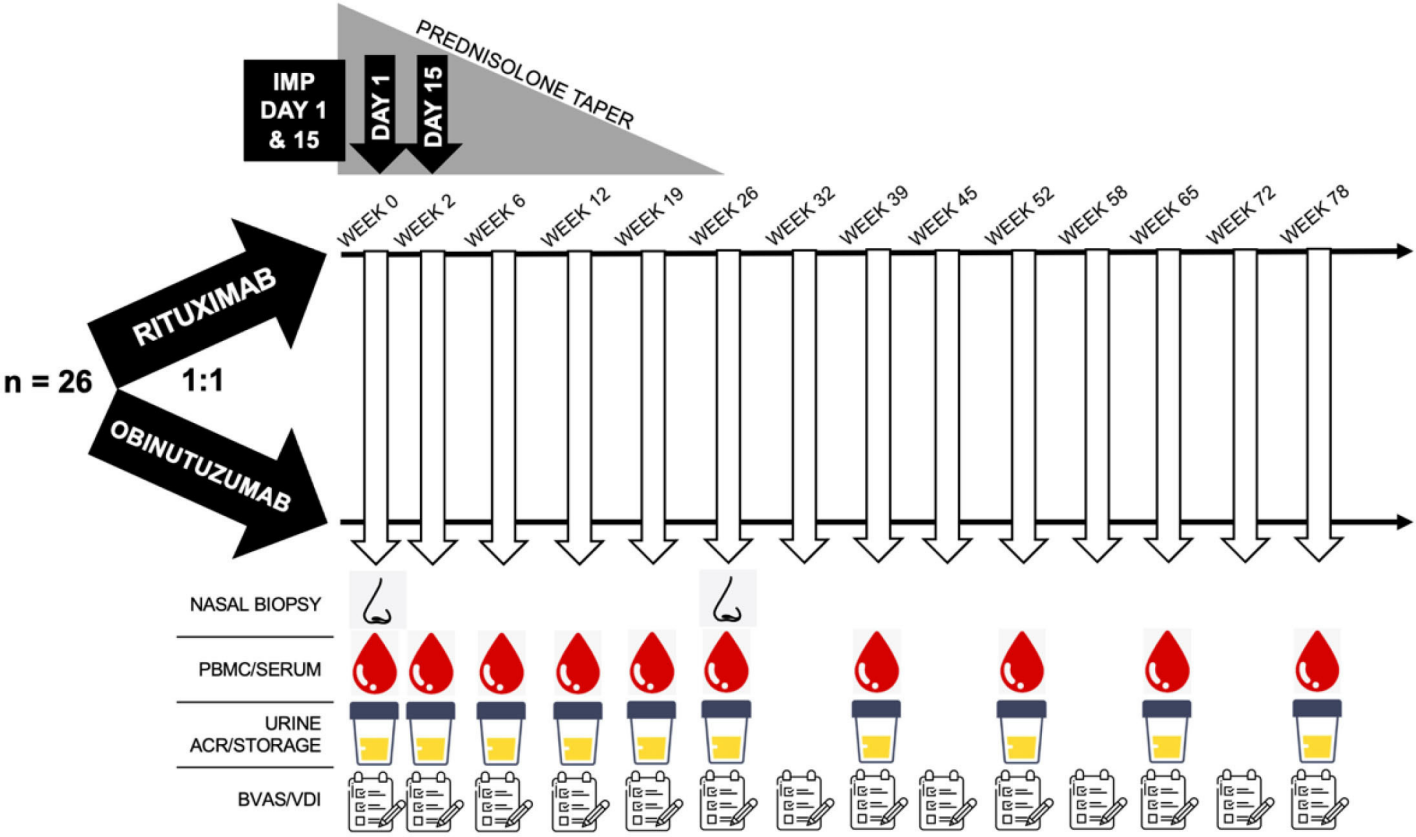
ObiVas trial schematic. On day 1, sample collection and assessments will be done before the first study dose. ACR, albumin:creatinine ratio; BVAS/WG, Birmingham Vasculitis Activity Score for Wegener’s Granulomatosis; IMP, investigational medicinal product; PBMC, peripheral blood mononuclear cells; VDI, Vasculitis Damage Index.

- Assessment of presence of germinal centres, T:B cell interactions, T cell activation status in the nasal tissue (spectral flow cytometry, single-cell RNA sequencing, confocal microscopy).
- Assessment of BCR repertoire/clonality analysis of the returning B cells in the blood at baseline, week 78 or relapse (BCR RNA sequencing).
- Assessment of BCR repertoire/clonality analysis of nasal tissue at baseline, and week 26 (BCR RNA sequencing).
- B cell functional assays at weeks 0, 52 and 78 (spectral flow cytometry).
- T cell functional assays at weeks 0, 12, 26, 52 and 78 (spectral flow cytometry).
- Serum will be collected and stored at multiple timepoints (figure 1) and may be used for other exploratory assays and post hoc analyses, such as IMP pharmacokinetics and antidrug antibodies.

Some of our exploratory outcomes will be assessed using next-generation sequencing technologies, including single-cell RNA sequencing. Such methodologies allow the study of immune mechanisms with a high level of granularity and approach the data in an unbiased manner, providing a greater insight into the multifaceted nature of tissue immunopathology in AAV, and how responses differ depending on the IMP.

AESIs include infections requiring antimicrobial, antiviral or antifungal treatment; hypogammaglobulinaemia; neutropaenia; thrombocytopaenia; systemic infusion reactions; hypersensitivity reactions; severe skin reactions; malignancy; cardiac disorders; thromboembolism; gastrointestinal perforation; pregnancy; abnormal liver function tests; overdose of IMP; suspected transmission of an infectious agent with IMP and lack of therapeutic efficacy of either IMP.

### Participant timeline

A schematic diagram summarising the proposed visits and interventions is displayed in figure 1. In addition to the NALT biopsies at baseline and week 26, participants will have routine clinical review, with formal assessment of disease activity using the BVAS-WG and Vasculitis Damage Index questionnaires. Serial peripheral blood mononuclear cell (PBMC) isolation for spectral flow cytometry will be performed. Urine and serum will be stored and may be used to validate the results of the exploratory outcomes (eg, cytokine validation of transcriptomic signals). The full schedule of activities is provided in online supplemental appendix 2.

### Recruitment

The participants will be recruited from the Vasculitis and Lupus Clinic at Addenbrooke’s Hospital. Prospective participants will be provided with a detailed patient information sheet (online supplemental appendix 3) and will be given time to review this and discuss with family and/or friends prior to providing informed written consent, which will be obtained by the investigator or the designee. Recruitment commenced in January 2023 following ethical and UK regulatory approval and at the time of publication, 18 participants have been randomised and received the IMP. The planned end date for recruitment is December 2024, with final trial follow-up for the last patient predicted to be in July 2026.

### Allocation

Eligible participants will be assigned to one of the two trial arms, either on day 1 or during the screening period once all eligibility criteria have been met. Randomisation must occur before IMP is administered using the online Sealed Envelope. Randomisation will be blinded and based on blocked randomisation, with stratification by prior rituximab use, using random block sizes. Participants will be randomised to obinutuzumab or rituximab in a 1:1 ratio.

### Blinding

Participants will be randomly assigned to receive obinutuzumab or rituximab in a double-blind fashion. Following randomisation, individual drug kit identification numbers will be generated for each participant and the trial pharmacy team will have a code list to convert the identification numbers into the unblinded treatment allocation. Obinutuzumab and rituximab will be identical in appearance, provided by the pharmacist with a blinded label and require the same premedications and infusion rate. Neither the investigator nor the participant will know which IMP is being administered.

In an emergency situation in which immediate knowledge of the administered IMP is required, the participant may be unblinded using Sealed Envelope.

### Participant withdrawal

Participants will have the option to withdraw from the trial and revoke their consent at any point. If consent to participate is withdrawn after receiving the IMP, permission will be sought by the investigators to allow them to collect efficacy and safety data from the participants’ electronic medical records until the end of the trial. Investigators may withdraw participants from the trial protocol before the administration of the IMP is completed for appropriate medical reasons (eg, SAE/reaction or pregnancy). Participants who withdraw consent—or are withdrawn by the investigator before IMP completion–will be encouraged to attend an early withdrawal visit.

### Data collection and management

In addition to the research samples outlined in the trial schematic (figure 1), participants will also have routine blood and urine tests, clinical and safety assessments recorded at each in-person assessment. After day 15, when the second IMP dose is administered, participants will be assessed by the trial team approximately every 1–2 months until the final trial visit at week 78. Following IMP administration, the key biomarker research assessments for the secondary and exploratory objectives (where blood will be gathered for transcriptomics and PBMC isolation and flow cytometry) will be at weeks 12, 26, 52, 65 and 78.

Clinical data will be anonymised and compiled on paper case report forms (CRF). The CRFs will be accessible to trial coordinators, data managers, the investigators, clinical trial monitors, auditors and inspectors as required. The paper CRFs will then be transcribed into a MACRO database. Research samples will be anonymised, logged and tracked and processed or stored in the University of Cambridge. Anonymised biomarker datasets will be stored on a secure server in the University of Cambridge. Any participant records or datasets transferred to the sponsor and/or the IMP manufacturer (Roche) will contain only the participant’s unique trial identification number and no other personally identifiable information.

Once the study team is made aware of an SAE, they must inform the Chief Investigator and report to the sponsor within 24 hours.

### Data analysis plan

Online supplemental appendix 4 describes the statistical analysis plan for ObiVas. The full analysis (FA) population will contain all patients randomised in the trial, while the PP population will exclude those who do not complete IMP treatment or are prescribed additional remission induction therapies. Patients with progressive disease before remission who receive additional treatment with additional glucocorticoids or avacopan will be included in the analysis. An avacopan-free population analysis will exclude patients who have received avacopan at any stage in their treatment, to assess if avacopan has any additional impact on lymphocyte depletion.

The primary analysis of this trial will compare the percentage change from baseline in nasal CD19^+^ B cells between day 1 and week 26 between obinutuzumab plus prednisolone (active) and rituximab plus prednisolone (control) in all randomised patients (expressed as means difference, SE, p value and 95% CI between the treatment groups). Analysis of covariance will be applied with fixed effects for treatment allocation at randomisation, baseline CD19^+^ B cells (continuous) and adjusted for by prior rituximab use (yes vs no).

Safety data incorporating incident rates of SAEs and AESIs and plots of incidence rate by treatment will be included. There will also be a summary of any adverse events causing IMP discontinuation.

A sensitivity analysis will be conducted which examines the differences between patients with and without a diagnosis of progressive disease, adjusting for whether they received avacopan during the trial. Furthermore, additional sensitivity analyses will be conducted using the primary efficacy analysis as a base case. Additional covariates will be fitted to the model such as prior cyclophosphamide use, age, sex and ethnicity to assess the additional impact, if any, of these covariates.

### Data monitoring

The Trial Steering Committee (TSC) will provide overall supervision of the study by monitoring its progress and ensuring that it completes within the agreed timescale and budget. The TSC includes three physicians with experience in clinical trials in vasculitis. The Data Safety Monitoring Committee will provide oversight for this trial and consists of three additional independent physicians with expertise in vasculitis trials and one independent statistician. They will meet and review unblinded safety reports every 6 months until all participants have completed the trial. They can make direct recommendations to the TSC as they deem appropriate.

## Ethics and dissemination

### Research ethics approval

Before the trial commenced, ethical approval was granted by the Health Research Authority (HRA) and Health and Care Research Wales (HCRW), following their review of the trial protocol, patient information sheet, informed consent form, GP letter and all other documents given to patients (eg, steroid tapering protocol). The Research Ethics Committee (REC) (Edgbaston (West Midlands)) approval reference is 22/WM/0174. Current protocol version is 4.0. An annual progress report will be submitted to the REC and they will also be required to approve any substantial protocol amendments.

### Consent

Participants will be required to provide written informed consent, which will be obtained by the principal investigator or appropriate trial delegate. Prior to this, participants will be provided with a Patient Information Sheet (PIS), which provides a detailed overview of the trial in lay terms. The PIS and informed consent form were approved by REC and are compliant with Good Clinical Practice, local regulatory requirements and legal requirements.

The informed consent also covers the storage of any biological samples for up to 1 year after the end of the trial. After this period, the samples will be transferred to a licenced storage facility (eg, tissue bank), used in a separate ethically approved research study or disposed of appropriately. Participants also provide their consent for the use of their anonymised data to be shared with third parties after the completion of the trial to facilitate further research. This inclusion of anonymised data in future data linkage studies is explained in the PIS (online supplemental appendix 3).

### Confidentiality

All gathered trial data will be stored in areas with limited access in locked file cabinets or in password-protected computer files. Patients will be assigned a trial identification number at trial entry, to limit the use of identifiable data. Any participant records or datasets that are transferred to the sponsor and/or IMP manufacturer, Roche (including SAE/SUSAR (suspected unexpected serious adverse reaction) reports) will contain the identifier only; participant names or any information which would make the participant identifiable will not be transferred.

### Ongoing access to data

Ownership of data gathered as part of this trial resides with the trial team. Anonymised data will be shared with the IMP manufacturer, Roche, at the end of the trial. Samples may be used in future ethically approved research studies and/or be placed in a Human Tissue Authority licensed tissue bank at the end of the study. Essential trial documentation will be archived for 5 years after the end of the trial.

### Dissemination policy

After trial completion, the trial results will be submitted and published in a peer-reviewed journal and presented at scientific meetings. Following the final analyses, the results will be shared with the patients either directly or via communication with vasculitis patient group(s) at meetings and through patient newsletters.

## supplementary material

10.1136/bmjopen-2023-083277 online supplemental file 1

10.1136/bmjopen-2023-083277 online supplemental file 2

10.1136/bmjopen-2023-083277 online supplemental file 3

10.1136/bmjopen-2023-083277 online supplemental file 4
