# Supplementary material for: Study protocol for a randomised, phase II, double-blind, experimental medicine study of obinutuzumab versus rituximab in ANCA-associated vasculitis: ObiVas
Source: BMJ Open. 2024 Jul 17;14(7):e083277. doi: 10.1136/bmjopen-2023-083277 (PMC11256062; doi:10.1136/bmjopen-2023-083277)
Supplement: online supplemental file 1 [file bmjopen-14-7-s001.pdf]

ObiVas

ObiVas Inclusion Criteria

Participants are eligible to be included in the trial only if all of the following criteria apply:

1. Capable of giving signed informed consent.
2. Participant must be >=18 years of age at the time of signing the informed consent form.
3. Have a diagnosis of AAV (granulomatosis with polyangiitis or microscopic polyangiitis), according to the definitions of the Chapel Hill Consensus Conference (35).
4. PR3 ANCA positivity by ELISA at screening.
5. Have active disease defined by one major or three minor disease activity items on the Birmingham Vasculitis Activity Score for Wegener's (BVAS/WG).
6. Women of child-bearing potential (WOCBP) must agree to use effective contraception methods and agree to follow these methods for at least 18 months after the last dose of rituximab or obinutuzumab.
7. Has received at least two doses of any COVID-19 vaccination or have a positive titre for antibodies to the COVID-19 spike protein, in keeping with prior infection.

ObiVas Exclusion Criteria

Exclusion criteria apply to the participant's condition at screening unless otherwise stated.
Participants are excluded from the trial if any of the following criteria apply:

1. Women who are pregnant, plan to become pregnant or breast feed during the trial.
2. Current participation in any other interventional treatment trials.
3. Compliance: is unlikely to comply with trial visits based on investigator judgment.
4. MPO ANCA or anti–GBM antibody positivity by ELISA during screening.
5. Presence of pulmonary haemorrhage with hypoxia.
6. Estimated glomerular filtration rate (eGFR) <15 ml/min/1.73m^2.
7. Symptomatic herpes zoster within 3 months of screening.
8. Evidence of active or latent tuberculosis (TB) determined by a positive (not indeterminate) QuantiFERON®-TB Gold test (or equivalent).
9. Known hypersensitivity or significant allergies to monoclonal antibodies (including IMPs or to any of the excipients, e.g. murine proteins)
10. Malignant neoplasm within 5 years (from screening) excluding basal cell or squamous cell carcinoma of the skin treated with local resection only or carcinoma in situ of the uterine cervix treated locally and without metastatic disease for 3 years.
11. A history of a primary immunodeficiency or severe immunocompromise
12. IgA deficiency (IgA < 0.1 g/L).
13. IgG deficiency (IgG < 4 g/L).
14. Neutrophils < 1.5 x 10^9 cells/L.
15. B cell lymphopenia at screening (total CD19+ count <0.1x10^9/L).
16. Alanine transferase (ALT) >2.5x upper limit of normal (ULN).

17. Active bleeding disorders, and/or inability to support interruption to anticoagulant or anti-platelet therapies for nasal biopsy.
18. Severe nasal deformity precluding endoscopic assessment/biopsy of postnasal space
19. Severe heart failure (New York Heart Association Class IV) or other severe, uncontrolled cardiac disease.
20. Have a history of a major organ transplant or hematopoietic stem cell/marrow transplant.
21. Have an acute or chronic infection requiring management as follows:

    • Currently on any treatment for a chronic infection such as pneumocystis, cytomegalovirus, herpes simplex virus, herpes zoster, or atypical mycobacteria
    • Hospitalisation **solely** for treatment of proven infection requiring parenteral (IV or IM) antibiotics (antibacterials, antivirals, antifungals, or anti-parasitic agents) within 60 days of Day 1. NB Hospitalisation for a participant with active vasculitis with co-existent infection requiring IV or IM antibiotics is permitted.
    • Proven severe infection requiring outpatient treatment with parenteral (IV or IM) antibiotics (antibacterials, antivirals, antifungals, or anti-parasitic agents) within 60 days of Day 1. Prophylactic anti-infective treatment is allowed. Precautionary PO/IV antibiotics in a participant with active vasculitis is permitted.
22. Positive human immunodeficiency virus (HIV) antibody test.
23. Positive serology for Hepatitis B (HB), defined as: (i) HB surface antigen positive (HBsAg+) OR (ii) HB core antibody positive (HBcAb+)*.
24. Positive Hepatitis C (HCV) antibody test.
25. Any additional contraindication for IMP treatment as per the SmPCs
26. Have clinical evidence of significant unstable or uncontrolled acute or chronic diseases not due to vasculitis which, in the opinion of the principal investigator, could confound the results of the trial or put the participant at undue risk.
27. Have a planned surgical procedure, laboratory abnormality, or condition that, in the opinion of the principal investigator, makes the participant unsuitable for the trial.
28. * Any positive hepatitis results will be reported to the appropriate authorities.

Prior/Concomitant Therapy:

29. Live vaccine(s) within 30 days prior to Day 1, or plans to receive live vaccines during the trial.
30. Have received any anti-CD20 (or any other B cell depleting therapies including alemtuzumab) within 12 months of Day 1.
31. Have received any of the following within 180 days of Day 1:
    • Cyclophosphamide
    • Belimumab
32. Have received any of the following within 90 days of Day 1:
    • Anti-TNF or anti-IL-6 therapy (e.g., adalimumab, etanercept, infliximab, tocilizumab),
    • Abatacept,
    • Interleukin-1 receptor antagonist (e.g., anakinra),
    • Intravenous immunoglobulin (IVIG),
    • Plasmapheresis, leukapheresis.
33. Have received any investigational agent (that is not approved for use in the UK) within 60 days of Day 1.
34. Have received emergency IV steroid >3g methylprednisolone between 30 days prior to Screening Visit and up to Day 1 (including Day 1).
