# Supplementary material for: Study protocol for a randomised, phase II, double-blind, experimental medicine study of obinutuzumab versus rituximab in ANCA-associated vasculitis: ObiVas
Source: BMJ Open. 2024 Jul 17;14(7):e083277. doi: 10.1136/bmjopen-2023-083277 (PMC11256062; doi:10.1136/bmjopen-2023-083277)
Supplement: online supplemental file 2 [file bmjopen-14-7-s002.pdf]

**Full Title:**	ObiVas: A randomised, phase II, double blind, controlled mechanistic study of obinutuzumab versus rituximab in ANCA-associated vasculitis
**Short Title:**	ObiVas
**ISRCTN Number:**	13069630

Statistical Analysis Plan Outline

(Based on ObiVas Protocol Version 3.0 19 Dec 2022)

SAP outline version 0.4 19th October 2023

ObiVas

**Chief Investigator**	Dr Rachel Jones	**SAP Author**	Dr Simon Bond
Signature		Signature	
Date		Date	

1. Summary of Statistics Considerations

Trial design
A randomised, phase II, double blind, controlled trial designed to evaluate the mechanistic effect of obinutuzumab versus rituximab in active AAV. Participants will be randomised to one of two treatment groups in a 1:1 ratio and receive obinutuzumab (2 x 1000 mg, two weeks apart) plus prednisolone or rituximab (2 x 1000 mg, two weeks apart) plus prednisolone.

All participants will undergo nasal biopsies at baseline and Week 26. Follow up will last for 18 months following entry into the study.
26 participants will be randomised and dosed with obinutuzumab/rituximab. Primary endpoint analysis will be performed after all participants have completed the 26-week biopsy and assessment.

Primary objective

To compare the differential effects of obinutuzumab versus rituximab to deplete nasal tissue B cells

Secondary objectives

To compare the differential effects of obinutuzumab versus rituximab on nasal tissue B and T cell subsets.
To compare the differential effects of obinutuzumab versus rituximab on blood B and T cell subsets.
To compare the differential effects of obinutuzumab versus rituximab on B cell depletion and reconstitution in blood.
To compare the differential effects of obinutuzumab versus rituximab on changes in PR3 ANCA.
To compare the clinical efficacy of obinutuzumab versus rituximab.
To compare the safety of obinutuzumab versus rituximab.

Exploratory objectives

To compare the differential effects of obinutuzumab versus rituximab on T-B cell cross talk in the nasal tissue.
To compare the differential effects of obinutuzumab versus rituximab on BCR repertoire.
To compare the differential effects of obinutuzumab versus rituximab on functionality of B and T cell in the blood.

Primary outcome measure

- Percentage change from baseline in nasal CD19+ B cells between Day 1 and Week 26

Secondary outcome measures

- Percentage change from baseline in nasal B and T cell subsets between Day 1 and Week 26
- Percentage change from Day 1 in blood B, T, NK cells and subsets of interest at Week 12, 26, 39, 52, 65 and 78
- Proportion of participants with detectable peripheral B cells at Week 12, 26, 39, 52, 65 and 78
- Proportion of participants with PR3 ANCA negativity at Week 12, 26, 39, 52, 65 and 78
- Time to PR3 ANCA rise
- Proportion of participants in sustained remission (relapse-free) at Weeks 12, 26, 39, 52, 65 and 78
- Time to remission
- Time to first relapse (as measured by BVAS/WG or use of prohibited vasculitis treatment), in those who have achieved remission
- Time to first major or second minor relapse (as measured by BVAS/WG or use of prohibited medication), in those who have achieved remission.
- Cumulative exposure to corticosteroids between groups
- Proportion of participants with serious adverse events (SAEs) Week 78.
- Incidence of SAEs.
- Incidence and severity of AEs of special interest (AESIs).

Exploratory outcome measures

- Assessment of presence of germinal centres, T-B-cell interaction, T-cell activation status in the nasal tissue
- Assessment of BCR repertoire / clonality analysis of the returning B-cells in the blood at baseline, week 78 or relapse.
- Assessment of BCR repertoire/clonality analysis of nasal tissue at baseline, and week 26
- B-cell functional assays at Weeks 0, 52 and 78.
- T-cell functional assays at Weeks 0, 12, 26, 52 and 78.

Method of randomisation

Eligible participants will be randomly assigned to obinutuzumab plus prednisolone or rituximab plus prednisolone in a 1:1 ratio based on blocked randomisation with a stratification factor of prior rituximab use, using random block sizes (carried out by Sealed Envelope).

Sample size

Based on pre-clinical data of obinutuzumab use in cynomolgus monkeys, we can expect the tissue B-cell depletion effect of obinutuzumab to be similar to that observed from rituximab-induced depletion of peripheral blood B-cells, i.e., percentage reductions of ~90-95% (to be conservative in our estimation we will use 80%). Our pilot data from 6 AAV patients shows rituximab-induced depletion of nasal CD-19 positive B-cells is 52.9% (standard deviation 14.5%). The statistical power was tabulated by considering mean values of 55% in the rituximab arm, and 75% and 80% in the obinutuzumab arm with an assumed common SD of 14.5%. The design calculations are shown in the table below:

Sample Size	55% vs 75%	55% vs 80%
20	83%	95%
26	92%	99%

Furthermore, based on prior experience it is anticipated that 10% (2-3 patients) will have treatment failure in the first three months with a drop out after randomisation withdrawal rate of <5% (0-1patient). Inadequate nasal sample acquisition rate is also expected to be low at <5% (1-2 patients). Therefore, a per protocol endpoint analysis will consist of 20-26 patients.

Analysis populations

- Full analysis (FA) population

The FA population is defined as all patients randomised in the trial, regardless of whether they actually received treatment.

- Per protocol (PP) population

The PP population will exclude patients who do not receive the full course of IMP or are prescribed additional therapies. Additionally, patients diagnosed with progressive disease will be removed from PP population.

- Avacopan free population

The avacopan free population will exclude patients who have been administered avacopan. The aim of this population is to assess any differences between the use of avacopan and steroids on lymphocytes and b-cell depletion.

Analysis methods

Efficacy analysis

The primary analysis of this trial will compare the percentage change from baseline in nasal CD19+ B cells between Day 1 and Week 26 between obinutuzumab plus prednisolone (active) and rituximab plus prednisolone (control) in all randomised patients (FA and PP). Analysis of covariance will be applied with fixed effects for treatment allocation at randomisation, baseline CD19+ B cells (continuous) and adjusted for by prior rituximab use (Yes vs No).

Safety analysis

The safety analyses will be conducted on the FA population. All safety parameters will be summarised. Summary tables will be presented for incidence rates (number of patients with at least one incidence) of symptoms/AEs, serious adverse events (SAEs). AE plots of incidence rate by treatment and relative risk (95% confidence interval) between the two groups will also be presented. Summary of AEs that caused the investigational medicinal product (IMP) discontinuation together with the detailed list of reasons will be presented.<br><br>**Sensitivity analysis**<br>A sensitivity analysis will be conducted which examines the differences between patients with and without a diagnosis of progressive disease, adjusting for whether they received avacopan during the trial. Furthermore, additional sensitivity analyses will be conducted using the primary efficacy analysis as a base case. Additional covariates will be fitted to the model such as prior cyclophosphamide use (Yes vs No), age (continuous), sex (Male vs Female) and ethnicity (White vs Other) to assess the additional impact, if any, of these covariates.
**Key outputs of primary analysis**<br>• Estimates of means difference, standard error, p-value, 95% confidence interval between the treatment groups<br>• Estimates of mean, standard error, 95% confidence interval for each treatment group<br>• No. of patients and reasons for treatment discontinuation by treatment allocation.
**Full SAP for the final report**<br>This SAP outline will be the base for developing the SAP for the DMC. The final SAP for the end of study report using the Cambridge Clinical Trials Unit SAP template.

Trial flow chart

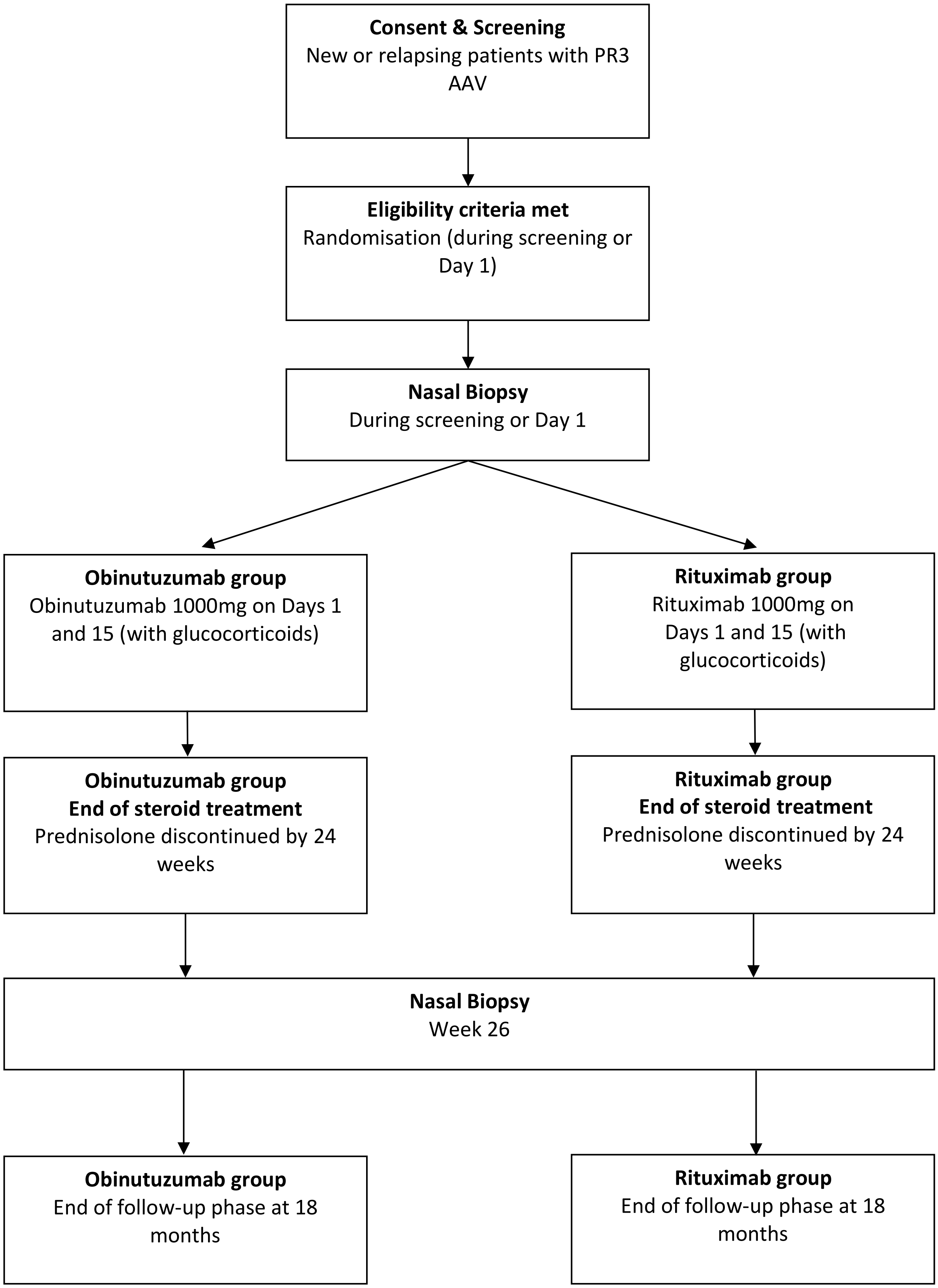

Screening period schedule of assessments

Procedure	Screening (up to 14 days before D1)
Informed consent	X
AE/SAE review (to be recorded from point of informed consent)	X
Inclusion and exclusion criteria	X
Demographics	X
**Assessments**	
Full physical examination including height and weight, vital signs (e.g. temperature, blood pressure, pulse)	X
Past and current medical conditions	X
12-lead ECG	X
BVAS/WG	X
**Laboratory Tests**	
PR3, ANCA, MPO ANCA, anti-GBM antibody	X
Serology (HIV antibody, hepatitis B surface antigen [HBsAg], hepatitis B core antibody [HbcAb] and hepatitis C virus antibody, SARS CoV-2 antibodies)	X
TB Quantiferon (or equivalent)	X
Immunoglobulins, Total CD19 count	X
FBC (Hb, WCC with differential, platelets), urea, creatinine, eGFR, sodium, potassium, ALT (or AST), ALP, bilirubin, albumin, CRP, ESR, glucose Urinalysis	X
INR and aPTT	X
Follicle-stimulating hormone (FSH) and oestradiol (women of non-childbearing potential only)	X
Serum human chorionic gonadotropin (hCG) pregnancy test (for women of childbearing potential)	X

Treatment and follow up period schedule of assessments

Trial Week[1]	Baseline	2 (Day 15)	6	12	19	26	32	39	45	52	58	65	72	78	Unscheduled visit[2,3,4]
Visit to trial centre[5]	X	X	X	X	X	X		X		X		X		X	X
Randomisation[6]	X														
IMP administration[7]	X	X													
AESI/SAE review[8,9]	X	X	X	X	X	X	X	X	X	X	X	X	X	X	X

[1] Phone calls to patients will be performed at weeks 32, 45, 58, and 72. If visits to the hospital are restricted (e.g. due to rise in Covid-19 infections rates) phone assessments can be performed instead of visit to trial centre when the only routine blood tests and a single research serum sample are required, and there is adequate mechanism in place for blood draw locally and shipment to site. Refer to the sample management manual for details.

[2] Unscheduled visits should be performed for relapse, safety concerns or early withdrawal from study.

[3] Research blood samples should be performed at unscheduled visits for relapse or early withdrawal from study (not for unscheduled visits for safety concerns).

[4] Participants withdrawing consent after randomisation, will be encouraged to complete an early Withdrawal visit.

[5] Visit for week 2 must occur within a +/- 3 day window of the scheduled visit. Visits for Week 6 to Week 52 must occur within a +/- 7 day window of the scheduled visit. Visits for Weeks 58 to 78 (Month 14 to Month 18) must occur within a +/- 14 day window of the scheduled visit.

[6] Randomisation can occur on D1 or during screening period after all eligibility criteria have been met.

[7] All blood/nasal samples must be taken before premedication and first obinutuzumab/rituximab infusion on day 1. Observation is required for 1 hour post IMP dose.

[8] Recording of all adverse events must start from the point of informed consent regardless of whether a participant has yet received a medicinal product.

[9] At Weeks 32 45, 58 and 72 when no visit is scheduled, the trial team must telephone the participants to check for AESIs/SAEs and if needed arrange an unscheduled visit.

Concomitant medications	X	X	X	X	X	X	X	X	X	X	X	X	X	X	X
Prednisolone schedule	X														
BVAS/WG	X	X	X	X	X	X		X		X		X		X	X
VDI	X					X				X				X	X
Medical Assessment	X	X	X	X	X	X		X		X		X		X	X
Routine labs[10],[11]	X	X	X	X	X	X		X		X		X		X	X
Urine pregnancy[12]	X													X	
Serum sample[13]	X	X	X	X	X	X		X		X		X		X	X
PBMC[14]	X			X		X		X		X		X		X	X
Whole blood transcriptomics	X			X		X				X		X		X	X

[10] Routine bloods include FBC (incl. WCC with differential), urea, creatinine, eGFR, sodium, potassium, bilirubin, ALT, ALP, CRP, ESR, glucose, immunoglobulins, PR3 ANCA

[11] Week 26 routine bloods should also include INR and aPTT (pre-biopsy).

[12] Urine pregnancy tests on day 1 and week 78 (or final visit) are only mandated for WOCBP.

[13] Serum in this study will be frozen for later use including but not limited to cytokine analyses.

[14] Flow cytometry will be performed and PBMCs will also be extracted from whole blood frozen (see sample management manual)

Nasal biopsy [15,16]	X					X									
Urine dipstick, UPCR, microscopy	X	X	X	X	X	X		X		X		X		X	X
Nasal swab	X					X									
Nasal activity score	X					x									
Urine sample storage[17]	X			X		X				X		X		X	X

[15] Baseline biopsies, nasal swabs and nasal activity scores can occur on D1 or during the screening period after all eligibility criteria have been met. Pre-biopsy work-up including recent (within 14 days) FBC/clotting screen must be performed before the biopsy. For the baseline biopsy, the FBC/clotting screen from the screening bloods may be used if within the 14 days. The Week 26 biopsy, swab and activity score should ideally occur on the same day as the Week 26 visit. FBC/clotting screen should be taken on the same day as the biopsy.

[16] If the biopsies are not performed on a scheduled trial visit day, an additional 9 mL research blood sample will be taken on day of biopsy.

[17] Urine samples will be stored as frozen (see sample management manual).
