# Supplementary material for: Study protocol for a randomised, phase II, double-blind, experimental medicine study of obinutuzumab versus rituximab in ANCA-associated vasculitis: ObiVas
Source: BMJ Open. 2024 Jul 17;14(7):e083277. doi: 10.1136/bmjopen-2023-083277 (PMC11256062; doi:10.1136/bmjopen-2023-083277)
Supplement: online supplemental file 3 [file bmjopen-14-7-s003.pdf]

Table 3 Treatment and follow up period schedule of assessments.

**Procedure**															
**Trial Week**[2]	**(Day 1) 0**	**(Day 15) 2**	**6**	**12**	**19**	**26**	**32**	**39**	**45**	**52**	**58**	**65**	**72**	**78**	**Unscheduled visit**[15,16,17]
Visit to trial centre[1]	X	X	X	X	X	X		X		X		X		X	X
Randomisation[3]	X														
Rituximab/Obinutuzumab[4]	X	X													
AESI/SAE review[5,6]	X	X	X	X	X	X	X	X	X	X	X	X	X	X	X
Con meds review	X	X	X	X	X	X	X	X	X	X	X	X	X	X	X
Prednisolone schedule given to patient	X														
BVAS/WG	X	X	X	X	X	X		X		X		X		X	X
Vasculitis Damage Index (VDI)	X					X				X				X	X
Symptom driven Medical Assessment (including vital signs)	X	X	X	X	X	X		X		X		X		X	X
Routine labs[7,8]	X	X	X	X	X	X		X		X		X		X	X
Urine pregnancy[9]	X													X	
Serum sample (frozen)[10]	X	X	X	X	X	X		X		X		X		X	X
PBMC (FACs and Frozen)[11]	X			X		X		X		X		X		X	X
Whole blood transcriptomics (frozen)	X			X		X				X		X		X	X
Nasal biopsy[12, 13]	X					X									
Urine dipstick, ACR, microscopy	X	X	X	X	X	X		X		X		X		X	X
Nasal Swab	X					X									
Nasal activity score	X					X									
Urine sample storage[14]	X			X		X				X		X		X	X

Footnotes

1. Phone calls to patients will be performed at weeks 32, 45, 58, and 72. If visits to the hospital are restricted (e.g. due to rise in Covid-19 infections rates) phone assessments can be performed instead of visit to trial centre when the only routine blood tests and a single research serum sample are required, and there is adequate mechanism in place for blood draw locally and shipment to site. Refer to the sample management manual for details.

2. Visit for week 2 must occur within a +/- 3 day window of the scheduled visit. Visits for Week 6 to Week 52 must occur within a +/- 7 day window of the scheduled visit. Visits for Weeks 58 to 78 (Month 14 to Month 18) must occur within a +/- 14 day window of the scheduled visit.

3. Randomisation can occur on D1 or during screening period after all eligibility criteria have been met.

4. All blood/nasal samples must be taken before premedication and first obinutuzumab/rituximab infusion on day 1. Observation is required for 1 hour post IMP dose.
5. Recording of all adverse events must start from the point of informed consent regardless of whether a participant has yet received a medicinal product.
6. At Weeks 32 45, 58 and 72 when no visit is scheduled, the trial team must telephone the participants to check for AESIs/SAEs and if needed arrange an unscheduled visit.
7. Routine bloods include FBC (incl. WCC with differential), urea, creatinine, eGFR (When the laboratory does not provide an eGFR calculation, a manual calculation will be performed using the same calculation), sodium, potassium, bilirubin, ALT, ALP, CRP, ESR, glucose, immunoglobulins, PR3 ANCA
8. Week 26 routine bloods should also include INR and aPTT (pre-biopsy).
9. Urine pregnancy tests on day 1 and week 78 (or final visit) are only mandated for WOCBP.
10. Serum in this study will be frozen for later use including but not limited to cytokine analyses.
11. Flow cytometry will be performed and PBMCs will also be extracted from whole blood frozen (see sample management manual).
12. Baseline biopsies, nasal swabs and nasal activity scores can occur on D1 or during the screening period after all eligibility criteria have been met. Pre-biopsy work-up including recent (within 14 days) FBC/clotting screen must be performed before the biopsy. For the baseline biopsy, the FBC/clotting screen from the screening bloods may be used if within the 14 days. The Week 26 biopsy, swab and activity score should ideally occur on the same day as the Week 26 visit. FBC/clotting screen should be taken on the same day as the biopsy.
13. If the biopsies are not performed on a scheduled trial visit day, an additional 9 mL research blood sample will be taken on day of biopsy.
14. Urine samples will be stored as frozen (see sample management manual).
15. Unscheduled visits should be performed for relapse/progressive disease, safety concerns or early withdrawal from study.
16. Research blood samples should be performed at unscheduled visits for relapse/progressive disease or early withdrawal from study (not for unscheduled visits for safety concerns).
17. Participants withdrawing consent after randomisation, will be encouraged to complete an early Withdrawal visit.
