# Supplementary material for: Study protocol for a randomised, phase II, double-blind, experimental medicine study of obinutuzumab versus rituximab in ANCA-associated vasculitis: ObiVas
Source: BMJ Open. 2024 Jul 17;14(7):e083277. doi: 10.1136/bmjopen-2023-083277 (PMC11256062; doi:10.1136/bmjopen-2023-083277)
Supplement: online supplemental file 4 [file bmjopen-14-7-s004.pdf]

ObiVas

PARTICIPANT INFORMATION SHEET & INFORMED CONSENT FORM

ObiVas: A randomised, phase II, double blind, controlled mechanistic study of obinutuzumab versus rituximab in ANCA-associated vasculitis

You are being invited to take part in a research trial. Before deciding whether to take part, you need to understand why this research is being done and what it involves. Please take time to read the following information carefully and talk to others about the trial if you wish. Please ask us if anything is not clear or if you would like more information. Please take time to decide whether or not you wish to take part.

Section 1 tells you why we are doing this trial and what taking part involves.
Section 2 gives you more detailed information about the conduct of the trial.

Section 1: Purpose of the trial and what will happen

1. What is the purpose of the trial?

In this trial, we want to test whether a drug called obinutuzumab works better than rituximab in treating the inflammation in your tissues and blood, which occurs when you have the medical condition called ANCA-associated vasculitis (Anti-Neutrophil Cytoplasmic Antibody – associated vasculitis). We want to see if obinutuzumab is more effective than a different drug called rituximab. Rituximab is the 'standard of care' drug, which means it is the treatment you would receive if you were not taking part in a trial.

2 What is the drug being tested?

The drugs being tested are obinutuzumab (also known as Gazyvaro®) and rituximab (also known as MabThera®).

ANCA-associated vasculitis is a disease in which the immune system (the system that fights infection) attacks your own cells, causing inflammation of the body's small blood vessels and tissues. It can affect almost any part of your body and involves antibodies (proteins made by the immune system in response to a foreign substance) called 'ANCAs'. ANCAs are produced by a type of white blood cell called B-cells. The drugs being tested (obinutuzumab and rituximab) both eliminate B-cells.

Rituximab is a standard treatment for vasculitis and although it works very well, repeated doses (typically every 6 months) are usually needed to keep the disease under control. Vasculitis tends to come back (also known as relapse) when rituximab is stopped and its effect wears off. Obinutuzumab is similar to rituximab, but it is thought that obinutuzumab is better at eliminating B cells in both the blood and the inflamed tissues (such as the nose). This may mean that vasculitis is less likely to relapse after obinutuzumab compared to rituximab and we want to test this in this study.

PARTICIPANT INFORMATION SHEET & INFORMED CONSENT FORM

ObiVas: A randomised, phase II, double blind, controlled mechanistic study of obinutuzumab versus rituximab in ANCA-associated vasculitis

Unlike rituximab, which is approved by regulatory agencies including the MHRA (Medicines and Healthcare products Regulatory Agency) in the UK to treat patients with ANCA-associated vasculitis, obinutuzumab is a newer drug that is not approved for vasculitis. However, like rituximab, obinutuzumab is approved for other medical conditions, including the B cell blood cancers (such as lymphoma). Other clinical trials are currently assessing whether obinutuzumab is an effective treatment for lupus (also known as systemic lupus erythematosus, or SLE), another autoimmune disease.

3 Why have I been invited?

You have been invited to participate in this trial because you have a particular type of vasculitis (proteinase 3 (PR3) ANCA-associated vasculitis) and it is currently causing you problems with your health and therefore you need to have treatment, such as rituximab.

In our study, we plan to include 26 patients with PR3 ANCA-associated vasculitis from Addenbrooke's Hospital, Cambridge.

4 Do I have to take part?

No - participating in this trial is voluntary. If you decide to be part of the trial, you will be asked to sign an Informed Consent Form; however, you will still be free to leave the trial at any point.

You can leave the trail at any time without giving a reason. However, it can be helpful to understand why people leave trials so you are welcome to tell us why. If you choose to leave the trial, your future medical treatment and normal medical care will not be affected in any way.

If we lose contact with you, but you haven't said that you wish to leave the trial, we will try to contact you by phone (no more than 3 times a week) for one month before taking you out of the trial.

5. What will happen to me if I take part?

If you agree to take part in the trial, you will be asked to sign the Informed Consent Form at the end of this document and will be given a copy of this to take away and keep for your own reference.

Before you start any treatment in the trial, we need to make sure that you are definitely able to take part. We will arrange a 'screening visit', where your trial doctor will ask you about your medical history and any medications you are taking. You will have a physical examination including vital signs (temperature, blood pressure, pulse)

and an ECG (electrocardiogram – a tracing of the heart). We will also take a blood sample (approximately 25 ml, equivalent to 2 tablespoons) and ask you for a urine sample.

Women who are unlikely to be able to bear children (i.e. women who have gone through the menopause) will have a blood test taken for follicle-stimulating hormone (FSH) levels, just to make sure there is no possibility they could bear children.

At screening, you will also have a blood test for infections such as hepatitis B and hepatitis C (viruses that cause inflammation of the liver), human immunodeficiency virus (HIV), and tests for previous or current tuberculosis (TB). If you take part in this study you will possibly need to have one chest X-ray. This is to rule out tuberculosis if the laboratory test is not available. You would not routinely need this if you were not taking part in the trial and had no chest symptoms (e.g., cough, breathlessness). This procedure uses ionising radiation but the dose is negligible, equivalent to around 3 days of average natural background radiation in the UK. Ionising radiation can cause cancer which manifests itself after many years or decades. The risk of developing cancer as a consequence of taking part in this study is between 1 in 5000 and 1 in 50,000, dependent on age, which is minimal. For comparison, the natural lifetime cancer incidence in the general population is about 50%. These are routine safety tests for any patient starting immunosuppressive treatments such as obinutuzumab or rituximab. Please note that we are not expecting positive results for these tests. However, if they are positive, we will make sure you are referred to the appropriate specialists. We are also required to report any new cases of hepatitis to the appropriate health authorities.

After screening tests, we will let you know if you are eligible for the trial. If you decide to participate, a computer will put you into one of the two treatment groups randomly. One of the groups will receive rituximab, whilst the other will receive obinutuzumab. Neither you nor your trial doctor can choose your group. This is called 'randomisation'. You have a 50% (i.e. 1 in 2) chance of being placed in either the rituximab or the obinutuzumab group.

Information about how the treatment that you receive is affecting your body and your health will be collected through a number of tests, procedures and questions over the course of the trial.

Double Blind:

This means that neither you nor your trial doctor will know which treatment you are receiving. However, your trial doctor can find out if it is absolutely necessary (i.e. in the case of an emergency).

PARTICIPANT INFORMATION SHEET & INFORMED CONSENT FORM

ObiVas: A randomised, phase II, double blind, controlled mechanistic study of obinutuzumab versus rituximab in ANCA-associated vasculitis

Treatments:

At the beginning of the trial you will receive an intravenous infusion (fluid drip into a vein) of either rituximab or obinutuzumab (1000 milligram (mg) dose is used for both drugs) on day 1 of the trial and then again on day 15. The infusion will be given by a trial nurse through a needle placed in a vein. Before receiving the infusions, you will receive medicines to reduce the risk of an allergic reaction. These will be an anti-histamine, a steroid and paracetamol (to prevent pain and fever). It takes at least 4-6 hours to give you the full infusion after which you will be observed for 1 hour to make sure you have no side effects.

At the beginning of the trial, you will also be given a course of steroid tablets (prednisolone) to help get your vasculitis symptoms under control. The dose will start at 60 mg per day and will gradually be reduced with a plan to stop by week 24. You will be given a prednisolone reduction schedule indicating when you should reduce your dose. The trial nurse will explain how to complete this and you should bring it with you to each visit. If you are on other immunosuppressants before entering the trial (e.g. methotrexate, azathioprine, or mycophenolate), these drugs will be stopped. This is standard practice but we will discuss this with you in more detail when you start the trial.

Nasal Biopsy samples:

On day 1 and at 26 weeks, you will undergo a biopsy, where we take a sample of tissue from the back of your nose. We will do this because vasculitis commonly causes inflammation of the inside of the nose and sinuses. The biopsies will allow the number and types of immune cells in your tissues to be looked at to see how active your vasculitis is and how well the drugs (rituximab or obinutuzumab) are working in your tissues.

The nasal biopsies will be performed under local anaesthetic by an experienced Ear Nose & Throat Surgeon in the outpatient clinic. The inside of your nose will be numbed with a local anaesthetic spray as well as with an anaesthetic-soaked gauze that will be put into your nostrils for 10 minutes before the procedure. This will make sure that the inside of your nose is completely numb and the procedure should not be painful. Two very small samples of tissue from the back of your nose will be taken at each biopsy. The procedure should take no longer than 20 minutes. Blood tests will be taken before the procedure to ensure it is safe for you to have the biopsy (see section 8 for possible risks of undergoing a nasal biopsy).

Follow Up:

PARTICIPANT INFORMATION SHEET & INFORMED CONSENT FORM

ObiVas: A randomised, phase II, double blind, controlled mechanistic study of obinutuzumab versus rituximab in ANCA-associated vasculitis

You will then have clinic visits approximately every 6 weeks for 6 months, and then every 12 weeks for 12 months (with a phone call in between visits to check how you are). That is 11 visits in total (including the initial screening visit) as well as 4 phone call assessments. You will also have a clinic visit if your vasculitis relapses during the trial.

At each visit, assessments including blood (approximately 50 ml or 3-4 tablespoons of blood) and urine samples, and vital signs including blood pressure, pulse rate and temperature measurements will be undertaken. These visits will be much shorter than the two visits for rituximab/obinutuzumab infusions or the nasal biopsy visits and will take approximately 15 minutes with a doctor and 30 minutes with a nurse.
At each visit, routine blood samples will also be taken to monitor your vasculitis as well as trial research blood samples. The doctors and nurses will check that your vasculitis is well controlled and that you are not having a relapse, in which case standard extra vasculitis treatments will be given to you. The research blood samples will be used to measure special markers of vasculitis activity and to measure the effects of rituximab/obinutuzumab on your immune system. The tests may also include looking at changes in ribonucleic acid (RNA), the genetic code that makes proteins in your body.

If you are female and are able to have children, urine pregnancy tests will be performed at screening, on day 1 and at 18 months, to make sure you are not pregnant before you start the trial and do not become pregnant during the trial.

During the course of the trial (18 months) the total amount of research and routine blood samples that will be taken will be about 520 ml (roughly 35 tablespoons).

All patients in this trial will have urine checks at each clinic visit for routine tests as well as specialist trial research tests. Approximately 100 mls (approximately half a cup) of urine will be collected 11 times during the trial for research tests.

PARTICIPANT INFORMATION SHEET & INFORMED CONSENT FORM

ObiVas: A randomised, phase II, double blind, controlled mechanistic study of obinutuzumab versus rituximab in ANCA-associated vasculitis

Assessments and visits table:

	Screening	Treatment		Follow up visits				
	Up to 14 days before Day 1	Day 1	Day 15	Week 6	Week 12	Weeks 19, 26, 39, 52	Weeks 32, 45, 58, 72 (telephone calls)	Weeks 65 and 78 (Months 15, 18)
Consent taken	X							
Pregnancy test*	X	X						X
Chest X-ray**	X							
Physical exam + vital signs	X	X		X	X	X		X
ECG	X							
Nasal biopsy		X				X (Week 26 only)		
Nasal swab		X				X (Week 26 only)		
Drug infusion		X	X					
Medication review	X	X	X	X	X	X	X	X
Blood sample	X	X	X	X	X	X		X
Urine sample	X	X	X	X	X	X		X
Assessments (BVAS/WG, VDI)***	X	X	X	X	X	X	X	X
Total time for visit	1 hr	8 hrs	6 hrs	45 mins	2 hr	45 mins	15 mins	45 mins

*only required for women of child bearing potential. A high follicle stimulating hormone (FSH) level will be taken to confirm a postmenopausal state in women not using hormonal contraception or hormonal replacement therapy.
**only required if alternate TB testing not available
***Your doctor will complete forms called ‘BVAS (Birmingham Vasculitis Activity Score)’ which is a scoring system to assess your vasculitis disease activity and VDI (Vasculitis Damage Index) to assess any organ damage since the onset of your vasculitis.

6. What will I have to do?

PARTICIPANT INFORMATION SHEET & INFORMED CONSENT FORM

ObiVas: A randomised, phase II, double blind, controlled mechanistic study of obinutuzumab versus rituximab in ANCA-associated vasculitis

Participation in this trial is for a year and a half (18 months). During this time, you will visit the clinic approximately 11 times. There will also be four scheduled phone calls. You should also tell the trial staff about any changes to your health in between visits. It is important that you take any trial medication e.g. steroid tablets (prednisolone) regularly, as directed by your trial doctor. You will also need to keep an accurate record of this on the prednisolone reduction schedule provided and return any unused prednisolone tablets to your trial doctor or nurse at week 24. If you are prescribed any medication by your GP you should also keep a record of this and let your trial nurse/doctor know.

What is the "Genetic Research" part of this trial and why is it being done?
The purpose of the genetics part of this trial is to see why different people may react differently to medicines. We get our genes from our parents, and different genes may affect who gets ANCA-associated vasculitis and related conditions or how the body reacts to a drug.
Scientists look for differences in people's genes that might explain this. Genetic research may include the study of certain genes or all your genes (also called your genome). This may include genes that affect how the drug works and how the body deals with the drug. It may also include genes linked to the immune system and conditions caused by it not working properly, such as ANCA-associated vasculitis.

How will being part of this trial affect my lifestyle?
Participation in this trial should not affect your lifestyle. The frequency of the trial clinic visits mean you will be coming to the outpatient's clinic a little more often than you would if you don't join the trial. Other than this, we encourage you to live your normal life, although you must not donate blood during the trial.

Trial medicines could harm an unborn baby or breastfeeding infant. You will not be able to take part in this trial if you are pregnant or breastfeeding. If you are female, you should not participate in this trial if you are planning to conceive a child during the trial or shortly afterwards. If you are male participant and have a female partner, she should not become pregnant for 3 months after your last trial infusion. Please share this information with your partner if it is appropriate.

Female participants who have child bearing potential must use one of the following, reliable forms of contraception for the entire trial (i.e. for 18 months after your last infusion with the trial drug (rituximab/obinutuzumab) :

- Oral contraceptive (either combined or progestogen alone)
- Contraceptive implant, injections or patches
- Vaginal ring
- Intrauterine device (IUD, coil or intrauterine system)

- Condom **and** cap or diaphragm **plus** spermicide (chemical that kills sperm)
- True abstinence where this reflects your usual and preferred lifestyle

You do not need to use contraception if:

- You are female and have only one male partner who has had a vasectomy (an operation to cut the tubes that carry sperm)
- You (or your partner) are a woman who cannot become pregnant
- You practice true abstinence as part of your usual and preferred lifestyle (no sexual activity from 30 days before the first infusion until 18 months (for women) or 3 months (for men) after the last infusion of trial medication). If you become sexually active, you must use one of the contraception methods listed above.

If you or your partner become pregnant during the trial or within 18 months of stopping treatment, you should let your trial doctor know immediately. Your trial doctor will discuss all the options available to you. The outcome and progress of any pregnancy would be followed and you would be asked questions about the pregnancy and baby, if appropriate.

What Medicines should I not take?

You should not take the following medicines during the trial:

- Any medicines that are part of other clinical trials
- Any immunosuppressive therapies (e.g. methotrexate, mycophenolate or azathioprine) unless recommended by your trial doctor for a relapse
- Live vaccines

Vaccines are designed to help prevent infections. After receiving obinutuzumab or rituximab, it is safe to have non-live vaccines (including influenza and COVID-19 vaccines) although they may not work as well as before receiving these treatments. Your trial doctor will review what vaccines you have had in the past and where possible any necessary non-live vaccinations will be given prior to trial start. You will not be able to receive a live vaccine such as BCG (for tuberculosis), oral polio, measles, rotavirus and yellow fever vaccinations while taking part in the trial as these are not safe in patients receiving rituximab or obinutuzumab.

If you have questions about any new medications or your current medications please talk to your trial doctor.

You should tell the trial team if you feel unwell or different in any way. If you have any concerns in between trial visits, please contact your trial doctor or nurse immediately using the contact numbers at the end of this information sheet.

If you take part in the trial, you should let any insurance provider you have (e.g. travel insurance, protection insurance, life insurance, income protection, critical illness cover and private medical insurance) know and seek advice if necessary. Failing to do discuss this with them could result in you being invalid for any future insurance claims.

7. What are the side effects of the drug being tested?

Like all medicines, rituximab and obinutuzumab can cause side effects, although not everybody gets them. Side effects may be mild or severe. We may give you medicine(s) to help lessen any side effects. Some side effects may go away as soon as you stop taking the trial drug. In some cases, side effects can be serious, long lasting or may never go away, or could rarely be fatal. If you are at all worried about your symptoms, then you can contact the trial doctor at any time (see contact name and number at the end of this leaflet). You may need to stop taking the trial drug after talking with the trial doctor.

Rituximab is considered 'standard treatment' for ANCA-associated vasculitis. This means that you are likely to receive rituximab as part of your routine medical care and that there is good knowledge about rituximab side effects in vasculitis patients. Current knowledge about obinutuzumab side effects comes from the experiences of other patients with different diseases, such as lymphoma, leukaemia and lupus. Reactions to the drug when it is being dripped in via a cannula (known as 'infusion reactions') and infection risks are the main concerns with both treatments. We want to test if obinutuzumab is better than rituximab at removing the B cells (part of your immune system) from your blood and tissue to see if it is better at treating ANCA-associated vasculitis. However, we also need these B cells to help fight infection. Therefore, in theory, there is a risk that infections may be more frequent after obinutuzumab than rituximab, but we will be monitoring closely for this – and other side effects – throughout the trial.

Infusion Reactions: Mild infusion reactions are common, but severe infusion reactions are rare. Infusions reactions are more common with the first infusion than the second infusion. During or within the first 24 hours of the rituximab or obinutuzumab infusion, you may develop fever, chills and shivering. Less frequently, some patients may experience pain at the infusion site, blisters, itching, sickness, tiredness, headache, breathing difficulties, tongue or throat swelling, an itchy or runny nose, vomiting, flushing or palpitations. If you have heart disease or angina, these reactions might get worse. The infusion may need to be slowed down or stopped. When these symptoms settle, the infusion can be continued.

Infections: Infections such as head colds, sinusitis, chest infections (e.g. pneumonia) and urine infections are common in vasculitis patients receiving immunosuppressants such as rituximab and obinutuzumab. Shingles may also occur. Rarely, serious infections (including bacteria, viruses and fungi) can occur. These can be life-threatening. As well as increasing the risk of infection, there is the possibility rituximab or obinutuzumab may reactivate old infections. Infections may be more common after obinutuzumab compared to rituximab. Infection rates will be monitored carefully during this trial.

Progressive Multifocal Leukoencephalopathy (PML): This is a very rare but severe viral infection, which causes brain damage and can be fatal. There is an increased risk of PML in patients receiving rituximab or obinutuzumab, although this is extremely rare. Tell your doctor if you develop trouble with memory loss, trouble thinking, and difficulty walking or sight loss.

COVID-19 infection and vaccination: Rituximab and obinutuzumab reduce the body's response to the COVID-19 vaccine, meaning that if people have a COVID-19 vaccine after receiving rituximab or obinutuzumab, the vaccine will work less well. However, rituximab or obinutuzumab should not affect the level of COVID-19 immunity that you have built up before starting these treatments. For this reason, participants should have had at least two COVID-19 vaccine doses, or have a positive titre for antibodies to the COVID-19 spike protein, in keeping with prior infection, before entering this trial.

People receiving rituximab who have not been vaccinated and catch COVID-19, are about 2-5 times more likely to have a severe illness and have a higher risk of death compared to people not taking rituximab. Following COVID-19 vaccination, rituximab patients are less likely to have a severe COVID-19 illness but their risk is still greater than people who have not received rituximab. Whilst this is a real concern for patients with vasculitis, this must be weighed against the benefits of properly treating vasculitis (which can be a life-threatening disease) and preventing vasculitis relapses. Vasculitis patients who have received rituximab or obinutuzumab who catch COVID-19 will be prioritised for access to NHS COVID-19 treatments.

Skin Reactions: Very rarely, after rituximab or obinutuzumab severe blistering skin conditions that can be life threatening may occur. Redness, often associated with blisters, may appear on the skin or on mucous membranes, such as inside the mouth, the genital areas or the eyelids, and fever may be present.

Other possible side effects associated with rituximab and obinutuzimab include:

Very common side effects (may affect more than 1 in 10 people):

- infections, such as colds, chest infections, urinary tract infections and herpes infections
- allergic reactions that are most likely to occur during an infusion, but can occur up-to 24-hours after infusion
- diarrhoea
- flu-like illness
- fevers
- blocked or runny nose
- muscle weakness
- decreased levels of natural antibodies (immunoglobulins) in the blood that fight infections

Common side effects (may affect up to 1 in 10 people):

- dizziness
- shakiness, often in the hands (tremors)
- indigestion
- constipation
- skin rashes, including acne or spots
- flushing or redness of the skin
- decreased number of red blood cells (anaemia)
- decreased numbers of platelets in the blood which may result in bleeding
- an increase in the amount of potassium in the blood (if left untreated, can become dangerous for the heart)
- changes in the rhythm of the heart, or the heart beating faster than normal
- low numbers of neutrophils (white blood cells that fights infection)
- swelling of the hands or ankles

Uncommon (may affect up to 1 in 100 people):

- nose bleeds
- painful joints
- muscle spasms
- difficulty sleeping (insomnia)

Rare (may affect up to 1 in 1000 people):

- chest pain, breathlessness, palpitations due to cardiac disorders (angina, atrial fibrillation, heart failure)

Very rare but serious side effects (may affect up to 1 in 10, 000 people):

- severe blistering skin conditions that can be life threatening. Redness, often associated with blisters, may appear on the skin or on mucous membranes, such as inside the mouth, the genital areas or the eyelids, and fever may be present
- recurrence of a previous hepatitis infection which can cause liver damage
- poor memory, confusion, unsteadiness and difficulty speaking can be signs of a severe brain infection called Progressive multifocal leukoencephalopathy (PML, see above) which can cause brain damage
- severe abdominal pain caused by a small hole in the wall of the bowel/gut, which very rarely occurs in patients with vasculitis affecting their gut
- confusion, seizures and drowsiness due to high blood pressure affecting the brain (posterior reversible encephalopathy PRES)

8. What are the possible disadvantages and risks of taking part?

In addition to side effects related to medications, when having blood tests, you may feel faint, or experience mild pain, bruising, irritation or redness at the site of the needle. In rare cases, you may get a skin infection.

Approximately 11 visits to the clinic and 4 phone calls are required over the 18 month trial period. This is more frequent than what is usually required in the standard treatment of vasculitis.

When deciding whether to take part in this trial, consider how the tests listed above and the visit schedule will affect your work and family schedules. Consider if you need transportation to get to the clinic. You may find that these tests and visits are inconvenient and require special effort. In addition, some tests may be uncomfortable.

Please ask us if you have any questions about the tests and procedures for the trial.

<u>What are the risks of a nasal biopsy?</u>

Small nasal biopsies performed under local anaesthetic by an experienced Ear Nose and Throat surgeon have a very low complication rate. The most common risks of having a nasal biopsy are minor bleeding and infection. Bleeding can be easily treated using gauze in the nose or by cauterising (matchstick-like instrument applied to small blood vessels in the nose to stop bleeding). The risk of infection is extremely low as sterile equipment is used and will be given some antibiotic cream to put in your nose after the procedure. Your nose may feel sensitive for a short while.

The most common risk of local anaesthetic is minor discomfort when injecting the inside of the nose. As with any medication, there is a small risk of an allergic response

to the anaesthetic. Other possible side effects include dizziness, nausea, vomiting, accelerated heart rate, slow heart rate and prolonged numbness.

9. What are the possible benefits of taking part?

There is no guarantee that you will benefit from taking part in this trial. You may experience relief in your symptoms or an improvement in your disease. However, information we collect from your urine, blood and tissue may benefit patients with ANCA-associated vasculitis in the future.

10. What are the alternatives for treatment?

Your trial doctor can tell you about other treatment choices for your ANCA-associated vasculitis. You may choose to:

- Continue to get regular care from your doctor
- Take part in another trial
- Get no treatment at this time

11. What happens when the trial stops?

Obinutuzumab is currently not an approved standard treatment for ANCA-associated vasculitis. If you were allocated to the obinutuzumab treatment group, you will not receive any more obinutuzumab when the trial stops. Standard vasculitis treatment will be continued as required, according to your local hospital practice.

12. Expenses & Payment?

You will not receive any payment for participating in this trial. However, you can receive reimbursement for any reasonable travel expenses and parking costs incurred for making extra visits to the hospital for this trial, which are in addition to your regular clinical visits. Please keep your receipts if you wish to do this.

Section 2: Trial Conduct

13. What if new information becomes available?

Sometimes during a trial, new information comes to light which might affect your decision to continue in the trial. Your trial doctor will contact you to discuss the new information and whether you wish to continue in the trial. If you still wish to continue in the trial, you will be asked to sign a new Informed Consent Form.

The trial sponsor (Cambridge University Hospitals and Cambridge University), the regulatory authority (MHRA, or Medicines and Healthcare products Regulatory Agency), the independent data monitoring committee or your trial doctor may decide

to stop the trial at any time. If that happens, we will tell you why the trial has been stopped and arrange for appropriate care and treatment for you.

14. What if I decide to change or stop my participation in the trial?

You are free to stop participating in this trial at any time without giving a reason and without it affecting your future medical care or treatment. If you decide not to participate any more, you will not receive any more trial infusions. No further tests will be performed on you and no further research samples will be collected. Any data or samples already collected or results from tests already performed on you or your samples will continue to be used in the trial analysis, unless you explicitly request otherwise.

The trial doctor may also choose to withdraw you from the trial if they feel it is in your best interests or if you have been unable to comply with the requirements of the trial. Reasons for trial withdrawal could include:

- You have experienced a serious side effect
- You are unable to complete the visits, or medication as required
- You become pregnant or plan to become pregnant
- Your trial doctor feels you no longer appear to be benefiting from the treatment

Should you experience serious side effects from the trial medication that requires you to withdraw from the trial, your trial doctor will follow-up with you regarding your progress until the side effect has stabilised or resolved.

15. What if there is a problem?

Any complaint about the way you have been dealt with during the trial or any possible harm you might suffer will be addressed. If you have any concerns about any aspect of this trial, you should speak to your trial doctor who will do their best to answer your questions.

In the event that something does go wrong, where you are harmed by taking part in the research and this is due to someone's negligence, then you may have grounds for legal action for compensation against Cambridge University Hospitals NHS Foundation Trust or the University of Cambridge. The normal National Health Service complaints mechanisms will still be available to you (if appropriate). The University has also obtained insurance that provides no-fault compensation (i.e. for harm not caused by negligence). The NHS does not provide no-fault compensation (i.e. for harm not caused by negligence), and NHS bodies are unable to agree in advance to pay compensation for non-negligent harm. They are able to consider an ex-gratia payment (i.e. gestures of goodwill) in the case of a claim.

If you wish to complain or have any concerns about any aspect of the way you have been approached or treated during this trial, you can do this through the NHS complaints procedure. In the first instance, it may be helpful to contact the Participant Advice and Liaison Service (PALS) at Addenbrooke's Hospital (see section 23 for contact details).

16. How will we use information about you?

Cambridge University Hospitals NHS Foundation Trust (CUH) and The University of Cambridge are the joint Sponsors for this clinical trial based at Addenbrooke's Hospital, Cambridge, UK.

We will need to use information from your medical records for this research project.

This information will include your NHS number, name and contact details. People will use this information to do the research or to check your records to make sure that the research is being done properly. If travel reimbursement is required, we shall take your bank details to be able to complete a bank transfer. This information will be kept in the strictest confidence and is only accessed by authorised members of the finance department.

People who do not need to know who you are will not be able to see your name or contact details. Your data will have a code number instead.

We will keep all information about you safe and secure.

Once we have finished the trial, we will keep some of the data so we can check the results. We will write our reports in a way that no one can work out that you took part in the trial.

De-identified information about your health and care may be made available for other research studies run by Cambridge University Hospitals and/or the University of Cambridge or other organisations. These organisations may be NHS or other public sector organisations, academic institutions, charities and commercial companies in the UK or abroad. Before your data is shared with other organisations, all personal identifiers (such as names, addresses and dates of birth) will be removed. Making information from trials available for further research helps maximise the benefit of your participation in the trial and allows other researchers to verify results and avoid duplicating research.

17. What are your choices about how your information is used if you leave the trial?

- You can stop being part of the trial at any time, without giving a reason, but we will keep information about you that we have already collected.
- We need to manage your records in specific ways for the research to be reliable. This means that we will not be able to let you see or change the data we hold about you.
- If you choose to stop taking part in the trial, we would like to continue collecting information about your health from central NHS records. If you do not want this to happen, tell us and we will stop.

18. Where can you find out more about how your information is used?

You can find out more about how we use your information

- at www.hra.nhs.uk/information-about-participants/
- our leaflet available from www.hra.nhs.uk/participantdataandresearch. Alternatively, please visit, for Cambridge University Hospitals NHS Foundation Trust: https://www.cuh.nhs.uk/participant-privacy/. For University of Cambridge: https://www.medschl.cam.ac.uk/research/information-governance or email The Information Governance team at: researchgovernance@medschl.cam.ac.uk
- by asking one of the research team refer/to/add contact details
- by sending an email to cuh.gdpr@nhs.net, or
- by ringing us on 01223 256141

19. What will happen to my samples?

If you take part in this trial, you will be asked to give blood, urine, and nasal biopsy samples for research tests. If you give permission, some tissue from other biopsies you may undergo as part of your routine clinical care (e.g. kidney, lung), may be analysed as well.

These samples will be used for various tests to assess the safety of you continuing to receive the trial treatments and the effects of the trial treatments on your vasculitis and the disease processes driving it.

Your samples may also be used by the trial team or shared by the trial team with other companies or universities to support other research in the future to better understand vasculitis, other diseases or conditions, or to further develop the trial drug or other drugs.

PARTICIPANT INFORMATION SHEET & INFORMED CONSENT FORM

ObiVas: A randomised, phase II, double blind, controlled mechanistic study of obinutuzumab versus rituximab in ANCA-associated vasculitis

Your blood, urine, nasal samples and other tissue samples will be given the same code as your other trial information and kept in locked storage within University of Cambridge for up to 1 year after the end of the trial for analysis. After this one-year period, your samples will either:

1. Be transferred to an approved licenced storage facility (e.g., tissue bank)
2. Be transferred for use in a separate ethically approved research study
3. Be disposed of in accordance with the UK Human Tissue Authority code of practice.

Anyone who works with your samples will hold the information and results in confidence.

Should you decide to withdraw from this trial early, unless explicitly told otherwise by you, any samples already collected will continue to be used in the trial analysis.

20. What will happen to the results of the trial?

When the results of this trial are available, they may be published in peer reviewed medical journals and used for medical presentations and conferences. They will also be published on the EU Clinical Trials Register website, a central registry for all clinical trials conducted in the EU. The results of the trial will be thoroughly de-identified and you will not be able to be identified from any of the data published. After this trial is complete, results from this study maybe combined with data from other similar studies to gain further understanding of how vasculitis treatments work.

The pharmaceutical company Roche will collect safety data during the trial including side effects from rituximab or obinutuzumab. This will include your date of birth and study code number. As the manufacturer of obinutuzumab and rituximab (MabThera), Roche are required to keep track of all the data produced from trials that use their drugs. They want to collect information on how well the drug works in different conditions and look for trends in side effects that may not be obvious from smaller studies.

If you would like to obtain a copy of the published results please contact your trial doctor directly who will be able to arrange this for you.

When the trial is complete and the results have been analysed, your study team will let you know how well the rituximab and obinutuzumab worked. Also, when the trial is finished and the results are available, your trial doctor can find out whether you received obinutuzumab or rituximab.

PARTICIPANT INFORMATION SHEET & INFORMED CONSENT FORM

ObiVas: A randomised, phase II, double blind, controlled mechanistic study of obinutuzumab versus rituximab in ANCA-associated vasculitis

21. Who is funding the trial?

The trial is being funded by the Medical Research Council (MRC). The pharmaceutical company Roche is providing the rituximab and obinutuzumab used in this trial.

22. Who has reviewed this trial?

All research within the NHS is reviewed by an independent group of people called a Research Ethics Committee, to protect your interests. This trial has been reviewed and given favourable opinion by (West Midlands - Edgbaston Research Ethics Committee). The Medicines and Healthcare products Regulatory Agency (MHRA) who are responsible for regulating medicines in the UK have also reviewed this trial.

23. Further information and contact details

You can talk with the trial doctor about any questions or concerns you have about the trial, or if you think you have been hurt from taking part in the trial or if you have any questions about side effects. Please call:-

Dr Rachel Jones: Telephone no. X at any time.
Nurse tbc: Telephone no. tbc

Your hospital's Patient Advice and Liaison Service (PALS) contact details are:
Email: X   Telephone no. X

<u>In the event of an emergency, please contact:</u>

If you require advice out of normal office hours, please call Addenbrooke's switchboard on X and ask to speak to the on call renal registrar.

ObiVas

PARTICIPANT INFORMATION SHEET & INFORMED CONSENT FORM

ObiVas: A randomised, phase II, double blind, controlled mechanistic study of obinutuzumab versus rituximab in ANCA-associated vasculitis

ADULT PARTICIPANT

Principal Investigator: Dr Rachel Jones
Participant Number:

If you agree with each sentence below, please initial the box **INITIALS**

1	I have read and understood the Participant Information Sheet version 4.1; dated 20th October 2023 for the above trial and I confirm that the trial procedures and information have been explained to me. I have had the opportunity to ask questions and I am satisfied with the answers and explanations provided.	
2	I understand that my participation in this trial is voluntary and that I am free to withdraw at any time, without giving a reason and without my medical care or legal rights being affected, and that any data and samples already collected or tests already performed will continue to be used in the trial as described in this information sheet.	
3	I agree to undergo the two nasal biopsy procedures as outlined in the participant information sheet.	
4	I understand that personal information about me will be collected and used in accordance with this information sheet. This information will be kept in the strictest confidence and I will not be identifiable in any results published.	
5	I understand that sections of my medical notes or information related directly to my participation in this trial may be looked at by research personnel, responsible individuals from the sponsor and regulatory authorities where it is relevant to my taking part in research and that they will keep my personal information confidential. I give permission for these individuals to have access to my records.	
6	I understand that my GP will be informed of my participation in this trial and sent details of the trial.	
7	I have read and understood the compensation arrangements for this trial as specified in the Participant Information Sheet.	
8	I understand that the doctors in charge of this trial may close the trial, or stop my participation in it at any time without my consent.	
9	I have read and understood my responsibilities for the trial including using appropriate contraception as listed in section 6.	
10	I understand that de-identified information collected about me may be used to support other research in the future, including research conducted by both commercial and non-commercial organisations in the UK and abroad.	
11	I consent to genetic analysis being performed on my blood and tissue samples and I understand that my de-identified genetic data may be	

PARTICIPANT INFORMATION SHEET & INFORMED CONSENT FORM

ObiVas: A randomised, phase II, double blind, controlled mechanistic study of obinutuzumab versus rituximab in ANCA-associated vasculitis

	uploaded onto a publically accessible database as a supplement to the publication of this study	
12	I understand that if I lose touch with the trial team and I have not explicitly expressed to be withdrawn, they will make efforts to contact me for one month (no more than 3 times a week) before being withdrawing me from the trial.	

		INITIAL
**OPTIONAL**<br>**If an optional clause is left blank/not initialled we will consider this to mean you have declined consent**		**YES**
13	I agree to allow tissue from any biopsy samples taken as part of my routine clinical care (e.g. kidney biopsy) to be used for ethically approved research purposes, as part of this trial. I understand that samples and results will be sent securely within the trial team.	
14	I agree to allow the use of my unused samples at the end of this study to be stored in a licenced storage facility (e.g. tissue bank) or be used for research purposes in other ethically approved studies	

I agree to participate in this trial:

________________ ________________________ __________

Name of participant Signature Date

________________ ________________________ __________

Name of person taking consent Signature Date

Time of Consent (24hr clock):

1 copy for the participant, 1 copy for the trial team, 1 copy for the patient’s medical notes.
