# Supplementary material for: Study protocol for a randomised, phase II, double-blind, experimental medicine study of obinutuzumab versus rituximab in ANCA-associated vasculitis: ObiVas
Source: BMJ Open. 2024 Jul 17;14(7):e083277. doi: 10.1136/bmjopen-2023-083277 (PMC11256062; doi:10.1136/bmjopen-2023-083277)
Supplement: Supplementary file 5 [file bmjopen-2023-083277.reviewer_comments.pdf]

PEER REVIEW HISTORY

BMJ Open publishes all reviews undertaken for accepted manuscripts. Reviewers are asked to complete a checklist review form (**http://bmjopen.bmj.com/site/about/resources/checklist.pdf**) and are provided with free text boxes to elaborate on their assessment. These free text comments are reproduced below.

ARTICLE DETAILS

**TITLE (PROVISIONAL)**	Study protocol for a randomised, phase II, double blind, experimental medicine study of obinutuzumab versus rituximab in ANCA-associated vasculitis: ObiVas
**AUTHORS**	McGovern, Dominic; McClure, Mark E.; Coates, Matthew; Bond, Simon; Martinez Del Pero, Marcos; Mynard, Kim; Lee, Jacinta; Smith, Rona M; Jayne, David R.; Clatworthy, Menna; Jones, Rachel B.; Investigators, ObiVas

VERSION 1 – REVIEW

**REVIEWER**	Mekinian, Arsène<br>AP-HP
**REVIEW RETURNED**	02-Apr-2024

**GENERAL COMMENTS**	very interesting and well written study<br>Authors could add the exact phenotype definition of differents B T NK cells analysis and also those of B autoreactive etc cells

**REVIEWER**	Hartinger, Jan Miroslav<br>Charles University and General University Hospital Prague
**REVIEW RETURNED**	07-Apr-2024

**GENERAL COMMENTS**	Thank you for giving me the opportunity to review the article describing protocol of interesting ObiVas study. The article describes the design of RCT that compares the effect of rituximab (RTX) vs obinutuzimab (OBI) on the depletion of nasal tissue B cells in nasal-associated lymphoid tissue biopsies in patients with anti-PR3 positive ANCA associated vasculitis (AAV). Furthermore, secondary outcomes are plasmatic B-cell depletion dynamics and clinical safety and efficacy aspects. The study is well designed with great emphasis on the disease pathogenesis and mechanism of treatment effect, nevertheless there is a drawback regarding the very limited focus on the drugs and their clinical pharmacology, especially pharmacokinetics (PK). This should be clearly mentioned in the study limitations if no other sample measurements for PK analysis will be provided:<br><br>Major comments:<br><br>- trogocytosis and RTX-CD20 internalization is thoroughly discussed in the introduction but RTX and OBI pharmacokinetic covariates are not discussed at all. Regarding monoclonal antibodies (MABs), several covariates can decrease their exposure which may translate into reduced treatment efficacy. In AAV patients with relatively low level of proteinuria especially anti-drug antibodies (ADA) are of importance as they can reduce the

	half-life of MABs substantially. In my experience (several dozens of treated and screened AAV patients) AAV up to 10% of patients develop ADA against RTX detectable 6th month after the last RTX dose. If OBI will be proven to be more effective – one of the reasons may be lower ADA production induction in this humanized MAB in comparison with chimeric RTX. As the numbers of patients in treatment arms are small the study is prone to be influenced by small number errors. Development of ADA in only few of the patients may even reverse the study results. This may be even more important when patients with former RTX treatment course are included as they might have already developed ADAs during the former treatment and therefore relapsed.<br>- Another covariate that slightly influences the overall exposure to RTX is body weight. As the study is designed according to local British guidelines and not according to SmPC where dose according to BSA is recommended (as well as in the RAVE study that authors cite in the introduction), the administered doses normalized to BSA od BW may largely differ between patients. Both of these drawbacks can be addressed by PK analysis measuring individual RTX and OBI exposures. If these limitations will not be addressed, they should be clearly discussed in the introduction and mentioned in the list of limitations. This is very detailed study with great focus on the pharmacodynamic and pathogenetic aspects. As the blood samples will be collected 10 times during the study period in each patient, why not measure also the drug levels and perform PK analysis and analysis of ADA occurrence?<br><br>Minor comments:<br><br>For effective searching for this paper keywords not included in the title will be more effective as now keywords and title partially doubles some words.

VERSION 1 – AUTHOR RESPONSE

Reviewer 1

Comments to the Author:
very interesting and well written study
Authors could add the exact phenotype definition of different B T NK cells analysis and also those of B autoreactive etc cells

Response:
Whilst not described in the manuscript, our novel spectral flow panel designed for this study comprises 34 fluorophores, which will allow us to investigate the impact of the IMP on various immune cell compartments both in nasal-associated lymphoid tissue (NALT) and peripheral blood. On p6 of the manuscript, we have detailed the exact phenotypic definition for the B cells of the primary endpoint. For the secondary endpoints, we have listed some of the antigens included on the spectral flow panel that will enable us to subset and perform functional assays on different immune cell compartments.

The isolation of anti-proteinase 3 specific autoreactive B cells is known to be challenging but has been performed by others successfully after optimisation1. In addition to performing spectral flow

cytometry on the day of collection, we are also freezing peripheral blood mononuclear cells (PMBCs) as specified in our patient visit summary and are keen to perform post-hoc analyses on autoreactive B cell dynamics. However, as work is ongoing to optimise this methodology in our lab, we have not included this in the planned protocol analyses for now.

Reviewer 2

Comments to the Author:
Thank you for giving me the opportunity to review the article describing protocol of interesting ObiVas study. The article describes the design of RCT that compares the effect of rituximab (RTX) vs obinutuzimab (OBI) on the depletion of nasal tissue B cells in nasal-associated lymphoid tissue biopsies in patients with anti-PR3 positive ANCA associated vasculitis (AAV). Furthermore, secondary outcomes are plasmatic B-cell depletion dynamics and clinical safety and efficacy aspects. The study is well designed with great emphasis on the disease pathogenesis and mechanism of treatment effect, nevertheless there is a drawback regarding the very limited focus on the drugs and their clinical pharmacology, especially pharmacokinetics (PK). This should be clearly mentioned in the study limitations if no other sample measurements for PK analysis will be provided:

Major comments:

- trogocytosis and RTX-CD20 internalization is thoroughly discussed in the introduction but RTX and OBI pharmacokinetic covariates are not discussed at all. Regarding monoclonal antibodies (MABs), several covariates can decrease their exposure which may translate into reduced treatment efficacy. In AAV patients with relatively low level of proteinuria especially anti-drug antibodies (ADA) are of importance as they can reduce the half-life of MABs substantially. In my experience (several dozens of treated and screened AAV patients) AAV up to 10% of patients develop ADA against RTX detectable 6th month after the last RTX dose. If OBI will be proven to be more effective – one of the reasons may be lower ADA production induction in this humanized MAB in comparison with chimeric RTX. As the numbers of patients in treatment arms are small the study is prone to be influenced by small number errors. Development of ADA in only few of the patients may even reverse the study results. This may be even more important when patients with former RTX treatment course are included as they might have already developed ADAs during the former treatment and therefore relapsed.
- Another covariate that slightly influences the overall exposure to RTX is body weight. As the study is designed according to local British guidelines and not according to SmPC where dose according to BSA is recommended (as well as in the RAVE study that authors cite in the introduction), the administered doses normalized to BSA od BW may largely differ between patients.
Both of these drawbacks can be addressed by PK analysis measuring individual RTX and OBI exposures. If these limitations will not be addressed, they should be clearly discussed in the introduction and mentioned in the list of limitations. This is very detailed study with great focus on the pharmacodynamic and pathogenetic aspects. As the blood samples will be collected 10 times during the study period in each patient, why not measure also the drug levels and perform PK analysis and analysis of ADA occurrence?

Minor comments:

For effective searching for this paper keywords not included in the title will be more effective as now keywords and title partially doubles some words.

Response:

We thank reviewer 2 for their thoughtful and detailed comments. We agree that pharmacokinetics should be considered in this phase II study, and as noted by the reviewer we are storing serum throughout the study - for analyses such as PK and ADA at trial end. We have added wording to the manuscript to state that PK/ADA maybe performed at study end. In our previous phase II study (COMBIVAS – NCT03967925)2, we performed PK analyses in all trial patients (N=30) and found that rituximab was consistently present in blood at low level at 3 months. In ObiVas we have selected 6 months as the time point for the second nasal biopsy (primary endpoint tissue B cell tissue analysis) to ensure the absence of Rituximab/Obinutuzumab in blood; recognising that the presence of drug may confound CD20 B cell detection. We have adjusted page 7 of the manuscript.

Finally, we have made changes to the keywords as suggested by the reviewer.

1. Cornec D, Berti A, Hummel A, Peikert T, Pers JO, Specks U. Identification and phenotyping of circulating autoreactive proteinase 3-specific B cells in patients with PR3-ANCA associated vasculitis and healthy controls. J Autoimmun. 2017;84:122-131.
2. McClure ME, Gopaluni S, Wason J, et al. A randomised study of rituximab and belimumab sequential therapy in PR3 ANCA-associated vasculitis (COMBIVAS): design of the study protocol. Trials. 2023;24(1):180.

VERSION 2 – REVIEW

**REVIEWER**	Mekinian, Arsène<br>AP-HP
**REVIEW RETURNED**	30-May-2024

**GENERAL COMMENTS**	ALL COMMENTS ARE OK
